# Mapping the Content Landscape of Self-transcendent Emotional Experiences with Thematic Analysis and Hierarchical Clustering

**DOI:** 10.1007/s42761-026-00356-x

**Published:** 2026-03-19

**Authors:** Tiago Bortolini, Maria Clara Laport, Giovanna Novaes Bortolini, Clarissa Alves de Sá, Ronald Fischer, Jorge Moll

**Affiliations:** 1 D’Or Institute for Research and Education, 22281-100 Rio de Janeiro, Brazil; 2 D’Or Institute for Research and Education, São Paulo, SP Brazil; 3 IDOR Pioneer Science Initiative, 22281-100 Rio de Janeiro, Brazil

**Keywords:** Self-transcendent emotions, Awe, Thematic analysis, Cluster analysis, Cross-cultural research

## Abstract

Self-transcendent emotions (STEs), including positive and threatening awe, compassion, admiration, elevation, gratitude, and love, expand one’s focus beyond the self and foster a sense of connection with something larger (e.g., people, nature, a higher power). Questions remain about the content structure that differentiates or unifies these emotions. We conducted four studies in Brazil, analyzing 1,683 narratives from the general population to clarify the nature and range of STEs in this underrepresented context. Study 1 developed and validated items measuring these emotions in Brazilian Portuguese, particularly critical for awe, as it lacks a direct translation. In Study 2, a bottom-up, qualitative template analysis from participants’ narratives yielded an initial coding template with 24 themes. Study 3 refined this template through qualitative coding, producing a final set of 34 unique themes. The theme of ‘connection with living forms’ emerged consistently across all STE narratives. Self-reported valence classifications indicated that several STEs’ experiences were ambivalent. In Study 4, a thematic hierarchical analysis revealed a robust link between positive and threatening awe, suggesting that these two variants share thematic elements compared to other STEs. Our qualitative narrative approach highlights STE’s thematical diversity and ambivalent valence, indicating that STEs do not form a homogeneous emotion category. Instead, our findings suggest that STEs are loosely connected to emotional reactions that share transcendental properties and are linked to cultural and social identities, but are likely driven by distinct affective processes.

**Supplementary Information:**

The online version contains supplementary material available at 10.1007/s42761-026-00356-x.

Self-transcendent emotions (STEs), including gratitude, compassion, admiration, awe, and moral elevation, are often described as a distinct family of positive emotions characterized by their outward-directed attention (Pizarro et al., 2021; Stellar et al., 2017). They can enhance an individual’s sense of connection to others, places, and abstract concepts, promoting prosocial behavior, social harmony, personal growth, and well-being (Algoe & Haidt, 2009; Keltner & Haidt, 2003; Schindler et al., 2013; Van Cappellen & Rimé, 2014).

Evolutionary theories propose that STEs may have developed as adaptive mechanisms promoting human cooperation and social cohesion, crucial for group stability and survival in complex social environments (Gorelik, 2016; Stellar et al., 2017). At the proximate level, STEs might extend neural systems associated with attachment, creating an ‘extended attachment system’ adapted for human emotional bonding beyond immediate kin to include broader communities and abstract ideas (Moll et al., 2006, 2008). Nevertheless, despite the recognized evolutionary and social significance of STEs, a detailed understanding of their common themes and interrelationships remains incomplete. This paper employs template analysis of Brazilian participants’ narratives to identify recurring content themes of self-transcendent emotions.

We focused on eight emotional categories: admiration, love, compassion, positive and threatening awe, moral elevation, gratitude, and being moved. Although not exhaustive, these categories were selected based on key criteria. First, they all share a central characteristic of STEs: being directed toward others and promoting connection (Pizarro et al., 2021; Stellar et al., 2017; Yaden et al., 2017). Second, these emotions are often discussed under the umbrella of self-transcendent emotions because of their outward-directed focus (e.g., Stellar et al., 2017; Van Cappellen & Rimé, 2014; Yaden et al., 2017).

Previous empirical research on STEs has largely relied on quantitative methods that often simplify or overlook the specific nuances and qualitative aspects of individual emotional experiences. For instance, Abatista and Cova (2023) employed quantitative dimension-reduction approaches to identify common characteristics across a range of positive emotions. However, these approaches do not capture the rich, context-specific qualities that individuals themselves articulate, which may not be included in the researcher-provided rating scales. Consequently, a qualitative, narrative-focused approach is necessary to deepen our understanding of how individuals uniquely experience and interpret STEs (Garcia-Romeu et al., 2015).

The experience of STEs is significantly shaped by cultural context, as documented, for example, in the distinction between positive and threatening awe. Awe is defined as an emotional response to something perceived as vast that exceeds one’s frame of reference and prompts a need for cognitive accommodation (Keltner & Haidt, 2003). When this vastness is accompanied by feelings of apprehension or fear, such as during overwhelming natural events, it can be termed “threatening awe” or “threat-awe” (Chaudhury et al., 2022; Gordon et al., 2017). Nakayama et al. (2020) found that Japanese individuals exhibit a higher propensity to experience this threat dimension, while Stellar et al. (2024) observed that Chinese participants reported more fear during awe experiences compared to their American counterparts.

Such cultural differences underscore the importance of context in shaping emotional experience, aligning with constructivist views that emotions arise through interactions between basic affective states, culturally derived concepts, and situational appraisals (Barrett, 2017; Mesquita et al., 2016). The constructivist view predicts significant variation across cultures in how people experience and perceive emotions. On the other hand, a universalist approach (Breugelmans & Poortinga, 2006) predicts broad cross-cultural consistency with recurring core features for self-transcendent emotions. To distinguish between these views, it is essential to develop validated, context-free STE items that allow participants to express their emotional experiences, ensuring authentic and culturally grounded insights (Taves et al., 2023; Wolf et al., 2023).

Brazil provides a particularly valuable context for examining STEs due to its rich cultural dynamics, shaped by Indigenous, African, and European influences, and characterized by strong collectivism and traditions of social connection (Matsumoto et al., 2008; Pilati & Fischer, 2025). These characteristics and the frequent associations of STEs with religious or spiritual experiences create an ideal setting to comprehensively explore these emotions (Preston & Shin, 20^2017^; Van Cappellen et al., 2013). Additionally, certain STEs, particularly awe, lack direct translations into Brazilian Portuguese, highlighting potential conceptual mismatches and underscoring the need for culturally sensitive methodologies in cross-cultural emotional research (Breugelmans & Poortinga, 2006; Fiske, 2020; Wierzbicka, 1999).

To fill these gaps and contribute to culturally inclusive research, we conducted a series of four studies employing qualitative and quantitative methodologies. Study 1 aimed to develop and validate culturally adapted open-ended narrative prompts in Brazilian Portuguese to facilitate natural, authentic descriptions of STEs. Building on these validated prompts, Studies 2 and 3 applied template analysis (Brooks et al., 2015), a qualitative method that systematically explores thematic patterns in open-ended responses. These studies also assessed emotional valence, providing insights into the positive, negative, or mixed affective qualities of STE experiences. Finally, Study 4 utilized quantitative clustering of the coded theme frequencies to assess structural relations among STE narratives.

## Study 1 – Validation of Items to Extract Experiences’ Narratives

### Method

#### Design

We build on the recently validated Inventory of Nonordinary Experiences (INOE) for Brazilian Portuguese (Fischer et al., 2025), which contains three STEs: awe (positive), compassion, and love. In this study, we expand the original instrument by validating items designed to elicit written narratives of five additional types of self-transcendent emotional experiences in Brazilian Portuguese: admiration, threatening awe, being moved, elevation, and gratitude. During the validation of the original INOE in Brazil (Fischer et al., 2025), which had a generic awe prompt, participants related no narratives of threatening flavors, even though threat flavoring is an important feature of awe (Keltner & Haidt, 2003). Therefore, we aimed to validate a new item explicitly targeting the threatening variant of awe. To distinguish this threatening version, we refer to the original INOE awe item as ‘Awe (positive)’. In the English-language literature, being moved—often discussed alongside kama muta (Cova & Deonna, 2014; Zickfeld, Schubert, Seibt, Blomster et al., 2019; Zickfeld, Schubert, Seibt & Fiske 2019)—refers to episodes of feeling emotionally touched in socially meaningful contexts, frequently accompanied (but not invariably) by somatic cues such as moist eyes/tears, chills, or warmth in the chest. For Brazilian Portuguese, “comoção” is the closest high-frequency term. Although “comoção” can denote widely shared distress in public discourse (e.g., comoção nacional; national commotion), it is also commonly used to describe being deeply touched/moved. We therefore use “being moved” as our cross-lingual label and define the construct by the touched/moved phenomenology, treating somatic markers as typical but optional (Table 1). This placement aligns with recent taxonomic work that groups moved/touched with social/self-transcendent emotions and emphasizes cross-label nuance (Abatista & Cova, 2023). Importantly, participants responded to Portuguese prompts describing each experience, and the intended interpretation was used solely for the Response Process Evaluation method (RPE; Wolf et al., 2023) comprehension judgments (see Data Analysis below and Table 1 for wording and sources).

To minimize fatigue, each participant provided narratives for up to two of the eight emotions. Responses were aggregated at the item level to assess comprehension.

### Participants

We recruited participants from the general population from an online panel (www.netquest.com). Participants were compensated with points within the Netquest platform, which they could exchange for various gifts. Inclusion criteria required participants to provide informed consent and be at least 18. Data collection occurred from September 2023 to January 2024. The project was approved by the Research Ethics Board of the D’Or Institute for Research and Education (65573322.6.0000.5249).

A total of 369 participants reported having at least one STE experience. The final dataset comprised 362 adults (51% female) aged between 18 and 72 years (M = 43.1, SD ± 12.78). We excluded participants who typed random characters or provided non-related responses for the narratives. On average, each participant’s total time commitment to complete the online questionnaire was 9.05 min. The sample size was determined based on previous studies using the RPE method (Fischer et al., 2025; Taves et al., 2023).

The demographic breakdown of the sample included participants from different social classes and educational backgrounds, reflecting a broad spectrum of socioeconomic statuses. A complete sociodemographic description is provided in Table S1.

### Materials and Procedure

Items were developed to elicit narratives for each of the emotions of interest. These items were validated using qualitative methods (Wolf et al., 2023). Items related to positive awe, compassion, and love had already been validated in Portuguese for the Brazilian INOE (Fischer et al., 2025). Here, we created and validated items for the additional STEs of interest (i.e., admiration, being moved, elevation, gratitude, and threat awe).

The questionnaire structure for item validation was similar to the one used by Taves et al. (2023) in the US and India, but we included some adaptations made in the previous Brazilian adaptation study (Fischer et al., 2025). First, participants were presented with items describing an emotional experience (e.g., “I had an experience that, despite being frightening, filled me with profound wonder and fascination.“). After reading each description, they were asked whether they had experienced that emotion (“Yes, I have experienced it,” “No, I have not experienced it,” or “I am not sure if I have experienced it”). Participants who responded affirmatively were asked to provide a narrative describing the relevant episode. Those who responded negatively or were unsure were asked to describe a hypothetical situation they believed matched the description provided.

Following this, participants answered two additional open-ended questions: one asking them to explain why they thought their narrative matched the emotion described and another asking them to paraphrase the original item. These follow-up questions were added to encourage participants to elaborate beyond the often very brief initial narratives and to provide clearer evidence of how they understood and interpreted the item. After that, they responded to a yes-or-no question about whether they believed they had understood the item correctly. Finally, they were given a multi-line input field to provide any comments about the questionnaire.

Sociodemographic data were acquired from the online panel company. Social class was evaluated using the “Brazilian Criteria” (Critério Brasil) questionnaire (Kamakura & Mazzon, 2013). This instrument assesses socioeconomic status by assigning points based on the presence and number of durable goods (such as televisions, refrigerators, and washing machines) and the household head’s educational attainment. The cumulative score generated from these indicators classifies households into six distinct categories—ranging from class A (highest socioeconomic level) to classes D/E (lowest socioeconomic level). This method, widely recognized in Brazilian market research and social sciences, provides a robust framework for delineating socioeconomic segments in the population.

#### Items Translation and Creation

Most of the target emotions in this study have direct, widely used Brazilian Portuguese equivalents: “amor” (love), “admiração” (admiration), “compaixão” (compassion), “elevação” (elevation), and “gratidão” (gratitude). For these terms with clear Portuguese equivalents, items were directly translated and reviewed by native-speaker researchers for general understanding.

Because Portuguese lacks a single canonical term for “awe,” we framed the construct with a set of close synonyms (deslumbramento, maravilhamento, fascínio) that emphasize wonder and vastness while avoiding other synonyms such as “espanto” or “assombro”, which in Brazilian usage frequently convey startle/negative affect. For being moved, we used the Brazilian words “tocado” (touched) and “comoção” (literally translated as “commotion” but also emphasizing the feeling of being moved).

For elevation, to maximize clarity and naturalness, we validated a descriptive narrative item—“I had an experience in which I witnessed someone perform a great act of kindness or charity towards another person.”—instead of a label-based alternative (“I had an experience in which I have seen an elevated act”), which does not convey the intended meaning in Brazilian Portuguese.

Our prompts for the narratives and the response process validation described below enabled participants to access and describe the intended experiences without relying on a single category name. This mitigates potential translation and label-specific bias.

### Data Analysis

The items were validated using the RPE method (Wolf et al., 2023). Items were tested and iteratively revised (if necessary) based on open-ended responses provided by participants. To assess item clarity and understanding, a team of four trained Brazilian coders independently determined whether participants understood each item as intended, using intended interpretations as a guide (Table 1). As in the original INOE, intended interpretations were based on dictionary definitions and previous theories. Each response was rated on a 5-point scale (1 = Understood, 2 = Probably Understood, 3 = Not Enough Information, 4 = Probably Not Understood, 5 = Not Understood), with coders encouraged to add comments explaining their classifications. Analyses included only participants who provided complete responses for at least one emotion; individuals who entered random characters or irrelevant content in the narrative sections were excluded. Hypothetical narratives from ‘No/Not sure’ respondents were also used for the RPE analysis to determine item comprehension.

**Table 1:** Intended interpretations used for response classification in the response process Evaluation, and the references from which they were derived

Target emotion (Portuguese translation)	Intended Interpretation	Main theoretical references
Admiration (Admiração)	An emotion that reflects respect, consideration, and veneration for others (adapted from Oxford Language Online).	Abatista & Cova, 2023.
Awe (positive)* (Deslumbramento; Fascínio; Maravilhamento)	An experience of something vast which challenges one’s ability to understand it or goes beyond what could have been imagined previously.	Taves et al., 2023.
Awe (threatening) (Deslumbramento; Fascínio; Maravilhamento)	Similar to the positive awe item but focusing on the ambivalence and the ‘flavor’ of threat that can compose an experience of awe.	Gordon et al., 2017; Keltner & Haidt, 2003
Being moved (Comoção)	Someone who is or has become emotionally or felt “touched”. It can involve specific physical perceptions (e.g., moist eyes, tears, chills) or not (adapted from Oxford Language Online).	Abatista & Cova, 2023; Zickfeld, Schubert, Seibt, Blomster et al., 2019
Compassion* (Compaixão)	A sympathetic pity and concern for the sufferings or misfortunes of others (adapted from Oxford Language Online)	Taves et al., 2023.
Elevation (Elevação)	Experiences in which someone demonstrates humanity’s ‘higher’ or ‘better nature’, being ‘virtuous” or performs something considered good for others (donations, charity, etc.). Virtuous = who possesses and cultivates qualities of virtue (‘moral, religious, social, etc.’) (adapted from Oxford Language Online) .	Abatista & Cova, 2023; Algoe & Haidt, 2009; Haidt, 2000
Gratitude (Gratidão)	Recognition by a person of someone who has provided them with a benefit, assistance, a favor, etc. (adapted from Oxford Languages Online)	Abatista & Cova, 2023; Algoe & Haidt, 2009
Love* (Amor)	A deep feeling of affection and attachment, which includes but is not limited to romantic feelings (adapted from Oxford Language online).	Taves et al., 2023.

We followed the Validation Score (VS) method proposed by Taves et al. (2023) to evaluate item comprehension. This method calculates the proportion of responses classified as understood (U) to the total of understood and not understood responses (U/(U + NU). Responses that were classified as containing not enough information for classification were ignored in this calculation. Recognizing the possibility of false positives (when participants incorrectly indicate they have experienced an emotion) or false negatives (when participants incorrectly indicate they have not experienced it), Taves et al. (2023) suggested two additional metrics. The Positive Proportion Understood (PPU) represents the proportion of “Yes” responses that were classified as understood (U_Yes/(U_Yes + NU_Yes), while the Negative Proportion Understood (NPU) measures the proportion of “No” responses that were understood (U_No/(U_No + NU_No).

An item was considered validated if the overall Validation Score (VS) was 80% or higher, based on responses from at least 20 participants who provided valid answers. Taves et al. (2023) suggested revising the item to improve clarity or providing specific examples if this criterion was not met. However, no specific guidelines were offered to determine when adjustments should be made or whether additional data should be collected before revising the item. We applied subjective cut-offs to guide our adaptation process. Specifically, if an item achieved at least 60% understanding in a sample of at least 10 participants and no major problems hindering understanding were apparent from the responses of these participants, it was retained and re-evaluated with another group of respondents to assess the stability of its comprehensibility. However, if the understanding rate fell below 60%, the item wording was revised based on available evidence to improve clarity, and the revised version was then retested.

### Results

 In total, 521 narratives were evaluated from 362 participants (mean narratives per participant = 1.44). For each target emotion, we tested a single narrative-elicitation prompt and iteratively revised wording when responses analyzed via RPE indicated misinterpretation (criterion VS ≥ 0.80). Probes (open-ended answer justification, paraphrase, comprehension check) were uniform across emotions and used solely for comprehension judgments. All items were considered valid (i.e., VS ≥ 80) in the first wording, except for Awe (positive), which needed two iterations, and the Awe-threat item, which required five iterations. Table 2 presents the validation scores for each item. Final wordings (PT-BR + English glossary) are reported in Table S2; all tested versions, along with their RPE metrics, are reported in Table S3.

**Table 2:** Validated items and validation scores

Item	# Item versions	*N*†	ExpU	ExpNU	3s	VS	PPU	NPU	IDK
Admiration	1	56	46	5	5	90.2	90.9	88.9	7
Awe (positive)*	2	42	24	2	16	92.3	94.1	88.9	6
Awe (threat)	5	34	20	5	9	80.0	76.9	83.3	6
Being moved	1	55	44	1	10	97.8	97.5	100.0	3
Compassion*	1	52	47	1	4	97.9	97.6	100.0	4
Elevation	1	56	41	2	13	95.3	100.0	71.4	1
Gratitude	1	49	42	3	4	93.3	92.3	100.0	8
Love*	1	53	39	4	10	90.7	100.0	60.0	6

*N *sample size for item validation; *ExpU *number of participants who reported having experienced the specific phenomenon and demonstrated a correct understanding of the corresponding item; *ExpNU* number of participants who reported having experienced the specific phenomenon but demonstrated a lack of understanding of the corresponding item; 3s = number of narratives without enough information; *VS* Validation Score; *PPU* positive proportion understood; *NPU* = negative proportion understood; † The total N contains all valid responses per item, value is larger than the sum of the Expu, Expnu and 3s columns due to the number of individuals without the experience. *Items validated as part of the Brazilian validation of the original Inventory of Nonordinary Experiences (INOE-BR; Fischer et al., 2025)

For the positive awe item, we began with a direct translation of the original INOE awe item (“I had an experience of fascination or wonder that stood out from all the other experiences of this kind I have ever had.“; “Eu tive uma experiência de fascínio ou maravilhamento que se destacou de todas as outras experiências do tipo que eu já tive.”). We observed that generic terms used to bridge the lexical gap for “awe” in Brazilian Portuguese (e.g., fascínio, maravilhamento, deslumbramento) sometimes steered respondents toward accounts of personal attainments or lifestyle changes rather than vastness that challenges prior understanding (e.g., “I was fascinated when I was able to choose my house.”). We therefore adopted a more descriptive wording emphasizing perceived grandeur and cognitive accommodation, which met the intended interpretation appropriately: “I had an experience in which I was fascinated and amazed to see, feel, or perceive something grand in a new way that challenged my previous thoughts; Eu tive uma experiência em que fiquei fascinado e maravilhado ao ver, sentir ou perceber algo grandioso de uma forma nova que desafiou como eu pensava até então.”. After this change, we obtained a greater variety of responses to the item, as well as narratives that reflected the intended interpretation (the complete dataset is available at the project OSF repository).

Because the positive-awe item elicited only positive narratives, we developed a dedicated item to capture awe experiences with threatening flavors. The first wording we tested (using perplexo / “perplexed/astonished”) was often interpreted as interpersonal surprise or dismay (e.g., “I went to a client’s house and she treated me really badly, even though I had already sold to this client several times”). Since this wording had a VS below the cut-off (Table S3), in the second wording we introduced the explicit threat qualifier (assustadora, “frightening”) to convey ambivalence while preserving fascination (“I had an experience that, although frightening, filled me with profound wonder and fascination”; “Eu tive uma experiência que, apesar de ser assustadora, me gerou profundo maravilhamento e fascínio”). Although the VS scores were promising, we decided to explore alternatives that could more explicitly highlight the threat component.

This generated wordings 3,4 and 5 (see Table S3) aimed at evoking more directly and specifically a flavor of threat in the experience, using words such as “medo” (fear), “ansiedade” (anxiety), “pavor” (dread), and “espanto” (which, in our sample, more commonly carried a connotation closer to fright than astonishment). However, these attempts led to interpretations more related to situations of pure fear (e.g., “I was robbed”) and various types of insecurity (“I met someone who asked me out, but I was afraid of getting involved. I knew he wanted something serious, and I wasn’t sure if that was what I wanted.”). Therefore, we decided to resume testing the second wording, which had shown promise. After a new wave of participants, it proved to be the most suitable of the five wordings, achieving a VS of 0.80. Unlike the general item, no detailed explanation of what is expected in an awe experience was needed.

Evaluating the narratives for item comprehension, we realized that determining the emotional valence was not possible from the narratives themselves. Although thematic analysis illuminates the qualitative essence of STE experiences, it does not always reveal whether these experiences’ valence is predominantly positive, as expected, negative, or mixed. For example, a narrative of compassion describing someone in need of help may not be clearly classified as positive, negative, or ambivalent. From a theoretical standpoint, valence measurement is crucial because it clarifies how individuals interpret and evaluate STEs — a particularly salient aspect given evidence of cultural variations in such appraisals (Chaudhury et al., 2022; Nakayama et al., 2020; Stellar et al., 2024). To address this gap, and in light of previous findings showing that cultural context can shape how people perceive and label STEs, we added a direct measure of emotional valence in Studies 2 and 3.

## Study 2 – Initial Coding Template of STE Narratives

### Method

#### Design

In this observational study, we utilized the items validated in Study 1 to explore the topics associated with participants’ written narratives of self-transcendent emotional experiences in an independent Brazilian sample. The study was conducted via online questionnaires, including open-ended and closed-ended questions to capture the richness and diversity of participants’ emotional experiences.

### Participants

In total, 478 participants from the same online panel used in Study 1 accessed the online questionnaire. The samples were completely independent. Participants were included in the final sample (*N* = 324) if they were over 18 years old, provided informed consent, reported experiencing at least one emotion, and provided high-quality responses (i.e., responses that were neither random nor unrelated to the task). Participants’ ages ranged from 18 to 79 (*M* = 45, *SD* ± 14), including 186 males and 138 females. Data collection occurred in February 2024. Complete sample demographics are detailed in Table S4. The Research Ethics Board of the D’Or Institute for Research and Education approved the project (65573322.6.0000.5249).

Considering the qualitative approach used in this study, no power analysis was used to estimate the sample size. The sample size was determined based on the available funding to recruit as many participants as possible from the online panel.

### Materials and Procedures

The online questionnaire’s structure was similar to that used in Study 1. Once giving consent, participants were randomly assigned to two possible branches comprising (1) items related to awe experience (positive or threatening) or (2) items related to experiences of the other six STEs. These two branches were created to avoid participant burden, as narratives of awe were followed by an additional questionnaire that is not reported in this paper. The validated items describing the emotional experiences (admiration, positive and threatening awe, being moved, compassion, elevation, gratitude, or love) were presented one at a time, and participants could answer whether they had had that experience or not (yes or no answer). If participants answered affirmatively, they were given a blank space to describe a situation in which they experienced the emotion. Following the narrative description, participants explained why they believed the reported experience was illustrative of the one described in the initial statement. These open-ended responses were used for the initial coding template analysis. Finally, they answered a multiple-choice item on the experience’s valence (i.e., positive, negative, both positive and negative, or neutral). Participants could view up to 8 items, and only those who reported experiencing at least one emotion were included in the final sample. We excluded participants after post hoc quality control for random or off-task responses. On average, participants spent approximately 7.11 min completing the survey.

### Data Analysis

Narrative responses were examined using template analysis (Brooks et al., 2015). Only narratives from participants who answered ‘Yes’ (i.e., reported having experienced the emotion) were included in qualitative coding and all downstream analyses.

The sample was divided among three independent coders, each responsible for classifying randomly assigned portions of the data. The coding was conducted blind to the narratives’ emotion categories, enabling the identification of shared themes across emotions. Each narrative could be assigned to as many themes as the coders deemed appropriate. Subthemes could be cross‑listed when indicated by content (e.g., grandchildren under Birth and Connection (Living Forms).

Our template was created using both inductive and theory-guided coding. We took steps to balance openness to the data with sensitivity to existing theories. Themes were initially generated and refined from the narratives in a bottom-up manner (inductive), without knowing which emotion category each narrative was supposed to represent. This blinding was intended to prevent coders from simply imposing expected features of, say, “gratitude” or “awe” onto the text. At the same time, the preliminary coding template included some theory-informed categories (deductive “seed” themes based on prior literature). In practice, if participants’ stories introduced a novel theme or nuance not anticipated by theory, it was added or split off as its own code; conversely, theoretically posited themes that failed to surface in the data were dropped or merged. This iterative template-analysis approach (Brooks et al., 2015) allowed us to capture unexpected, culturally specific content while still observing broadly theorized elements where they genuinely appeared.

Following the initial coding, the primary coder (MC) reviewed all responses and the assigned themes. This coder refined and reorganized the coding template to ensure it accurately captured the data and created subthemes where necessary to describe the narratives better. The coding template was then discussed with all coders to reach a consensus and finalize the structure. Study 3 will report results on the multiple-choice item measuring the experience’s valence since both studies used the same item.

### Results

Three hundred twenty-four participants reported experiencing at least one of the presented emotions, contributing an average of 1.82 narratives per participant. This resulted in 591 valid narratives, distributed as follows: admiration (*n* = 80), positive awe (*n* = 63), threatening awe (*n* = 62), being moved (*n* = 76), compassion (*n* = 73), elevation (*n* = 61), gratitude (*n* = 79), and love (*n* = 97).

The initial coding template, developed through an iterative process of template analysis (Brooks et al., 2015), identified 24 preliminary unique themes, reflecting substantial heterogeneity in content. The most frequent themes were Love (11.0%), Emotional bond (7.7%), and Prosocial behavior (7.1%), followed by Virtue (6.7%), Inequality (5.7%), Religion (5.6%), Spirituality (5.4%), Tragedy (4.9%), Birth (4.8%), Receive help (4.7%), and Achievement/Unprecedented (each 4.2%), among others (full list in Table S5). These themes span social, moral, spiritual, and adverse-event contexts, indicating a rich thematic diversity in Study 2 that is further refined in Study 3. Detailed theme descriptions, per-emotion distributions, and the interpretive synthesis are presented in Study 3.

These findings underscore the richness and variety of experiences associated with STEs, laying the groundwork for Study 3, which focused on refining and applying the coding template to the whole dataset. This allowed for a more thorough exploration of recurring themes across different individuals and contexts, providing a deeper understanding of the shared and unique elements of STEs.

## Study 3 – Refining the Template Coding

### Method

#### Design

This study had the same observational design as in Study 2.

### Participants

The sample comprised 466 participants who accessed the online questionnaire, 453 of whom completed it successfully and had experienced at least one of the emotions. There was no overlap with participants from Studies 1 and 2. Participants ranged from 18 to 76 years old (*M* = 43, *SD* ± 13), with 185 males and 268 females. The average time required for completing the online questionnaire was 9.85 min. Inclusion criteria required participants to provide informed consent and be at least 18. We excluded participants after post hoc quality control for random or off-task responses. Data collection occurred in June and July 2024. The research ethics board of the D’Or Institute for Research and Education approved the project (65573322.6.0000.5249).

### Materials and Procedures

The online questionnaire followed the same structure as in Study 2. Once giving consent, participants were randomly assigned to two possible branches comprising (1) items related to awe experience (positive or threatening) or (2) items related to experiences of the other six STEs. These two branches were created to avoid participant burden since narratives of awe were followed by an additional questionnaire not reported in this paper.

Additionally, two closed-ended questions about participants’ religiosity were presented at the end. After identifying the recurrence of religious-related themes in the first sample, we added a question to evaluate potential relationships between religious affiliation and the identified themes. Participants were asked about their religious affiliation, including “other” (see Table S4 for all categories). If “other” was selected, participants were provided a text entry to specify their religious affiliation. For those who chose any option other than “No religion,” a final multiple-choice question was asked concerning the importance of their religious affiliation in their life (“very important,” “somewhat important,” “not so important,” “not important at all,” and “I don’t know”; (Haerpfer et al., 2020).

### Data Analysis

As in Study 2, only those who reported experiencing at least one emotion were included in the final sample. We began by applying the initial coding template from Study 2 to the narratives. In line with standard template analysis procedures, this iterative step allowed us to refine the preliminary set of themes based on the new data. Whenever the data did not align with the existing themes, we adjusted the template by adding new themes, redefining existing ones, or removing redundant codes.

The Study 3 dataset was divided between two independent coders. Each coded a random subset of the data. All coders convened once the coding was complete to reach a consensus and ensure that the final template accurately captured the range of participant responses.

Once the final version of the template was established, we applied it to the complete datasets from Studies 2 and 3. This iterative process enabled further modifications, such as incorporating additional themes or revising existing ones, where the original template did not fully account for the diversity of responses.

Following this template analysis, we performed several quantitative analyses on the entire dataset to examine the distribution and prevalence of themes across various emotions. First, we identified the most frequently occurring themes that accounted for 90% of the sample. Next, we investigated the distribution of these themes within each specific emotion. Finally, we calculated the frequency and percentage for each theme for each emotion, allowing us to determine which themes were more strongly associated with specific emotional experiences.

#### Self-reported Valence of Experiences

Valence was recorded as Positive, Negative, Ambivalent, or Neutral to index appraisal type. Participants had to classify their experiences by selecting a single option. We modeled valence using a multinomial logistic regression (nnet::multinom) with emotion (8 levels) as the sole predictor. We computed marginal predicted probabilities (and 95% confidence intervals via parametric bootstrap from the model’s asymptotic covariance) for each Emotion × Valence cell.

We conducted two families of planned comparisons on the probability scale (all with Benjamini–Hochberg FDR control within family): (1) Within-emotion pairwise differences among the four valence categories (6 contrasts per emotion); and (2) Between-emotion pairwise differences across emotions (28 contrasts per valence).

We report percentage-point differences, 95% CIs, and FDR-adjusted p-values (Benjamini–Hochberg) for each family. Only narratives from participants who answered “Yes” (experienced the emotion) were analyzed.

#### Quote Selection

**Fig. 1:**
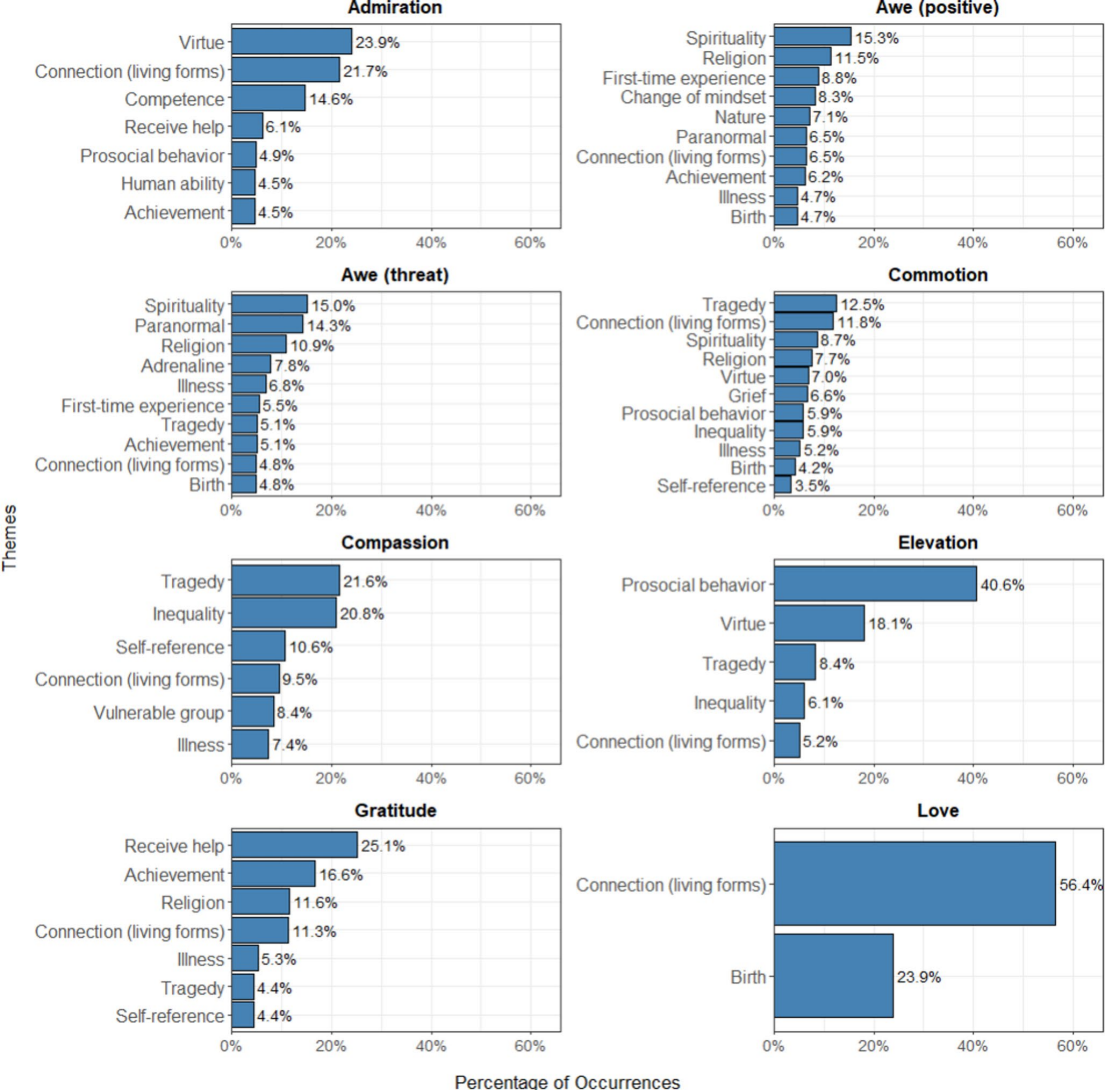
Distribution of Top Themes by Emotion. Each subplot displays the most frequent themes, accounting for approximately 80% of the total theme occurrences within each emotional category. This threshold was chosen for illustrative purposes and reveals the diversity of dominant themes across different emotional experiences. Only narratives from participants who reported having experienced the emotion (“Yes” response) were coded. Each narrative could receive multiple themes; percentages reflect within-emotion shares

To illustrate the qualitative findings, we included excerpts from participants’ narratives in Table S7. For each emotion, the main coder selected a quote that exemplified the highest-frequency themes, covering ~ 80% of within-emotion theme assignments (see Fig. 1). All original narratives and their corresponding codes are available in the project’s OSF repository.

## Results

Four hundred sixty-six participants reported experiencing at least one of the eight target emotions, yielding 1,045 valid narratives (*M* = 2.31 per participant). The distribution of these narratives across emotions was as follows: admiration (*n* = 119), positive awe (*n* = 132), threatening awe (*n* = 113), being moved (*n* = 112), compassion (*n* = 142), elevation (*n* = 117), gratitude (*n* = 131), and love (*n* = 179).

### Refinement of the Coding Template on the Study 3 Sample

In Study 3, we refined the Study 2 codebook by consolidating overlapping labels, disambiguating broad categories, and sharpening definitions where heterogeneous exemplars had been pooled. Out of 2,587 assigned themes, 772 were recoded (29.8%). Most of these changes involved merging the preliminary themes Love and Emotional bond into Connection (living forms), which accounted for 49.1% of all recoding. This step helped disambiguate the theme from the emotion label “love” and unified narratives emphasizing relational content. Along the same lines, other narratives originally coded as Love were redistributed according to their context or target—for instance, to Spirituality (1.68%), Connection (social group; 0.39%), Self-reference (0.26%), and Illness, Parenting, Prosocial behavior, or Others (all representing 0.13% of changes).

Several additional systematic refinements accounted for most of the remaining changes. The label NOE (non-ordinary experience) was reworded as Paranormal (7.51%) to provide terminological precision. Specific Groups was renamed Vulnerable group (7.25%) to better capture narratives involving marginalized populations. The Unprecedented theme was divided into First-time experience (6.99%) and Change of mindset (5.96%), distinguishing situational novelty from cognitive reappraisal. The broad theme Sensitivity was unpacked into Empathy (4.02%), Prosocial behavior (0.52%), and Virtue (1.17%), separating vicarious feeling, other-benefiting action, and agent-level traits. Similarly, the generic Connection code was disambiguated by referent: interpersonal/animal ties (Connection – living forms, 1.68%), transcendent ties (Spirituality, 1.42%), and community belonging (Connection – social group, 0.13%).

9.21% of changes involved narratives originally labeled Gratitude, which were re-categorized according to the dominant situational or target theme. For example, Religion or Spirituality when gratitude was directed toward God, Connection (living forms) when it centered on a relationship, or event-based categories such as Achievement, Illness, or Tragedy. Finally, a couple of recodes were done for themes with overall low occurrence (≤ 2), which were recoded as “Others” (Beauty, Nostalgia, Sacrifice, Shame, and Disappointment). The complete documentation of changes (Preliminary theme → Final theme) is reported in Supplementary Table S6.

### Template Analysis – Coding of all Narratives

#### Main Themes Across All Emotions

Once the revised coding template was finalized through consensus discussions among independent coders, it was reapplied to all Study 2 and Study 3 narratives. In total, 1,679 narratives were classified according to this final template. Overall, 30 unique themes were identified, with 18 representing 90% of the total themes. The five most frequently occurring themes were connection (living forms) (16.35%), Prosocial behavior (7.34%), Tragedy (7.34%), Virtue (6.73%), and Religion (6.11%), which together accounted for 44.04% of the total themes identified in the narratives. Table 3 presents the complete list of themes, their frequency of occurrence, and descriptions. The presence of religious themes among the top five most frequent themes may indicate sample bias, given that the majority of individuals in the sample were religious (Fig. S1A). However, the themes of Religion and Spirituality accounted for only 10.1% of the themes assigned to narratives by religious participants and 2% of the themes assigned to non-religious participants (Fig. S2).

**Table 3:** Themes descriptions and percentage of occurrence within the whole sample (*N* = 1683 narratives).

Theme	Description	Occurrence (%)	Cumulative (%)	Representative narrative
Connection (living forms)	Experiences involving emotional connections with living beings, such as people and animals.	16.35%	16.35%	“I adopted two girls, and I love them.” (*Love*)
Prosocial behavior	Narratives involving acts of kindness, altruism, or helping others, focusing on the actions taken to benefit someone else.	7.34%	23.70%	“Some people went to Rio Grande do Sul to help both the people and the animals that went through the tragedy. I admire them deeply and wish I had the resources to have gone as well.” (*Elevation*)
Tragedy	Stories involving catastrophic events or misfortunes that elicit strong emotional responses due to their severity and impact.	7.34%	31.04%	“I needed help to identify my deceased mother’s body because I had COVID.” (*Gratitude*)
Virtue	Admiration for individuals possessing benevolence or charitable qualities, focusing on their virtuous character (different from prosocial behavior, which emphasizes the act).	6.73%	37.77%	“I met a person who, regardless of their financial situation, never let anyone who came to their door leave without being fed, helping people in any circumstance.” (*Elevation*)
Religion	Experiences directly related to religious beliefs, practices, rituals, or events within an organized faith.	6.11%	43.87%	“The baptism with the Holy Spirit of God. It is indescribable, unique, and completely individual. Only those who have been reached by love will understand!” (*Awe positive*)
Spirituality	Experiences involving a sense of connection with a higher power, the universe, nature, or one’s inner self, not necessarily in the context of organized religion.	5.91%	49.79%	“I had a spiritual experience in which I was touched by someone who wasn’t there.” (*Awe threat)*
Receive help	Experiences where an individual receives assistance during a time of need, highlighting others’ support.	5.72%	55.51%	“When I was about to undergo an important surgery, a co-worker came to offer me financial help to pay for the procedure.” (*Gratitude*)
Birth	Narratives involving childbirth or the arrival of new life.	5.37%	60.88%	“The birth of my son was a mixture of happiness and the challenge of something new; even today it is difficult to explain the feeling.” (*Awe positive)*
Inequality	Narratives that highlight social disparities and injustices.	4.87%	65.75%	“When I see people sleeping on the sidewalks, it makes me very sad, because a human being living in such a difficult situation, without even the basics to live with a little dignity.” (*Compassion*)
Illness	Stories related to personal or others’ health challenges.	4.60%	70.35%	“My husband took care of his mother with cancer with great dedication.” (*Admiration*)
Achievement	Experiences related to accomplishing significant goals or reaching important milestones.	4.48%	74.84%	“When I finished my college degree, which was partly during the pandemic, with many difficulties and managing to pay for it on my own.” (*Gratitude*)
Self-reference	Instances where individuals relate others’ situations to themselves, projecting personal feelings or reflections.	2.98%	77.81%	“When I saw a patient at my work who was the same age as me in the terminal stage of intestinal cancer. He was the same age as I am, had similar interests to mine, and no prospects of life. And I kept wondering why he was in that situation and not me.” (*Compassion*)
Paranormal	Experiences involving supernatural or unexplained phenomena that challenge conventional understanding.	2.82%	80.63%	“I was driving and there was oil on the road, and the car skidded. As it spun on the road, an image appeared as if it were in my place, helping me not to overturn, controlling the steering. It was incredible, I stopped near a crater.” (*Awe threat*)
Vulnerable group	Experiences highlighting the challenges faced by marginalized or disadvantaged groups (encompassing animals).	2.44%	83.07%	“I once met an elderly woman in the subway. She told me that she lived alone, had no more family, no acquaintances, and that she was afraid of dying in a place where no one would find her. That was why she spent her afternoons in the subway, because it was well populated.” (*Being moved*)
Competence	Admiration for someone’s skill, expertise, or proficiency in a particular area, highlighting their capabilities and achievements.	2.24%	85.31%	“I saw a person standing out greatly in the field I intend to work in, which is international relations, acting both in the public and private sectors, in companies, and carrying out numerous mediations that were favored by the skills they had acquired through their training.” (*Admiration)*
First-time experience	Experiences focused on doing something for the first time, emphasizing novelty and new experiences.	2.16%	87.48%	“When I was a child, the first time I saw the planet Saturn through a telescope.” (*Awe positive*)
Change of mindset	Experiences involving a fundamental shift in one’s perspective, whereby previously held assumptions or beliefs are re-evaluated.	1.93%	89.41%	“I saw some lights in the sky that moved in unusual ways and stayed in the same place for more than an hour, which changed the way I think about whether we are alone in the universe.” (*Awe positive)*
Grief	Emotional experiences centered around loss and mourning.	1.86%	91.26%	“It was when I became a widow that many people helped me when I needed it the most.” (*Gratitude*)
Empathy	Experiences involving the ability to understand and share the feelings of others.	1.24%	92.50%	“One day my neighbor’s son went missing, and for months I saw her crying in the yard of her house. This made me feel her pain.” (*Compassion*)
Nature	Experiences involving a connection with the natural world, appreciating its beauty, serenity, or power.	1.24%	93.74%	“The waters reaching my city were frightening, but at the same time they caused me amazement; even though the lagoon was very full, the landscape was beautiful.” (*Awe threat*)
Human ability	Admiration for remarkable feats or abilities demonstrated by individuals or humanity as a whole.	1.20%	94.94%	“When I saw the pyramids of Egypt, it was simply spectacular considering the time in which they were built, the era, and the resources available.” (*Awe positive*)
Adrenaline	Experiences that elicit an adrenaline rush, such as engaging in extreme activities or thrilling situations.	1.12%	96.06%	“On the roller coaster descent at 120 km per hour, I was with a stranger because my husband didn’t want to go with me. I was afraid, but amazed by the sensation of speed, it was incredible.” (*Awe threat*)
Parenting	Narratives centered around the experiences of being a parent or caregiver, not related to childbirth.	0.93%	96.98%	“I never, under any circumstances, saw myself as a mother. Today I believe I have surpassed my own expectations. Being a mother is so rewarding. It changes all our thoughts, but for the better. A child is not an obstacle. It is exactly what we need at that moment in our lives.” (*Awe positive*)
Others	Experiences that do not fit into any other theme.	0.73%	97.72%	“I did things I hate for someone to make her happy. In love, you sacrifice yourself for the other person to the point of making them happy.” (*Love*)
Physiological response	Experiences characterized by intense physical sensations, such as goosebumps, chills, or an accelerated heartbeat.	0.62%	97.60%	“When I felt that I was about to faint, everything closing in, my vision narrowing, the sensation that nothing would exist anymore, that there would only be darkness; as if I were falling into a limbo, as if all my strength were disappearing…” (*Awe threat*)
Violence	Narratives involving acts of physical aggression, abuse, or harm.	0.54%	98.14%	“When I saw a woman being stabbed right in front of me.” (*Compassion*)
Suffering	Narratives centered around enduring pain, hardship, or adversity, highlighting the emotional toll of difficult experiences.	0.46%	99.07%	“It was an experience in which I saw a person I am not close to suffering because of offenses from others. I felt bad for that person, as it was not something pleasant to hear.” (*Compassion*)
Festivities	Narratives centered around celebrations, festivals, or joyous events.	0.31%	99.38%	“When I was the best man at a close friend’s wedding, I was very moved during the ceremony, especially because I had witnessed the couple’s difficult journey and was there to see their happy ending.” (*Being moved*)
Loss	Experiences involving the absence or deprivation of someone or something significant.	0.19%	99.57%	“I deeply admire my father’s strength. When we lost my mother, he found strength he didn’t even have to comfort his children.” (*Admiration*)
Connection (social group)	Experiences involving emotional ties within social groups or communities emphasizing a sense of belonging.	0.15%	99.73%	“Love for our homeland. If we do not have love and appreciation for our homeland, everything becomes bad and negative.” (*Being moved)*

#### Main Themes Per Emotion

The overarching theme encompassing the narratives of all emotions was the connection with other people or animals. This was the only theme to appear among the most frequent themes across all emotion categories (Fig. 1). Apart from this theme, the themes derived from narratives for each emotion differed substantially. The exemptions were positive and threat-related awe, which shared several common themes. Below, we summarize the main themes per emotion (see also Table 2 for theme definitions, frequencies, and representative narratives).

#### Admiration

Narratives of admiration most frequently described individuals displaying remarkable competence, where participants detailed witnessing exceptional skills or achievements, or acts of kindness.

#### Awe (Positive and Threatening)

Accounts of positive and threatening awe often involved experiences tied to organized religious practices, general spiritual experiences, and first-time experiences. In threatening awe, experiences were marked by an unsettling or fear-tinged quality, such as sensing a powerful force or presence that felt beyond ordinary human comprehension, or a rush of physical and emotional stimulation reminiscent of “adrenaline.”

#### Being Moved

Narratives often revolved around personally or collectively distressing events, such as accidents, disasters, or crises. Religious themes and spiritual themes, unaffiliated with a specific religion, were also common. Compared with typical English-language datasets, which are often predominantly positive and feature love/relationship elicitors (Cova & Deonna, 2014; Menninghaus et al., 2015), our narratives for being moved show a high proportion of ambivalent/negative themes and more references to collectively distressing events. We interpret this divergence as context-sensitive, reflecting contemporaneous collective hardship during data collection (e.g., flood-related news coverage affecting millions of people during the data collection). Crucially, even when negatively toned, Connection (living forms) emerged in references to those affected by the events, consistent with positioning being moved among social/self-transcendent states rather than mere sadness.

#### Compassion

Stories frequently revolved around Suffering, Tragedy, and Inequality, highlighting sensitivity to others’ hardship (e.g., illness, poverty, discrimination). Many accounts foregrounded the witness’s emotional resonance and concern; some included themes of received help or prosocial behavior, though immediate action was not always emphasized.

#### Elevation

Narratives prominently featured Prosocial behavior and Virtue, often in the face of adversity, portraying moral beauty and altruism. These accounts commonly linked admiration of others’ goodness with a felt motivation to do good, with Connection (living forms) present when ties to beneficiaries or observers were salient.

#### Gratitude

Participants most often described receiving help in challenging contexts, complemented by Achievement (e.g., completing a significant goal supported by others). References to Religion/Spirituality were also frequent when gratitude was directed toward God or a higher power.

#### Love

Narratives converged strongly on Connection (living forms)—enduring affection and care for partners, family, friends, or pets. Accounts of Birth were common, emphasizing the transformative impact of welcoming a child or a new family member.

#### Self-Reported Valence of Experiences

A multinomial logistic regression (nnet::multinom) with valence (Positive, Ambivalent, Negative, Neutral) as outcome and emotion (8 levels) as predictor fit the data substantially better than an intercept-only model (likelihood-ratio test: LR = 621.29, *df* = 21, *p* < 0.001). Information criteria favored the full model [AIC (full) = 2863.4, BIC (full) = 3003.9; AIC (null) = 3442.7, BIC (null) = 3460.2], and the pseudo-R² indicated an improvement in explanatory power (McFadden R² = 0.18; adjusted McFadden = 0.17). Marginal predicted probabilities are reported in Table S8. All subsequent inferences are based on model-based marginal probabilities and Benjamini–Hochberg–adjusted contrasts reported above.

##### Within-emotion Contrasts

Across all eight emotions, Positive appraisals were consistently the most likely. For threatening awe and compassion, Ambivalent responses were clearly elevated relative to Neutral and Negative, yielding more mixed profiles despite Positive remaining highest. Commotion showed the same pattern: both Ambivalent and Negative were higher than Neutral, with Ambivalent and Negative essentially similar. For positive awe, Ambivalent was higher than both Neutral and Negative; Negative and Neutral were comparable (*p* = 0.056). Elevation was overwhelmingly positive; Ambivalent exceeded Negative, Ambivalent and Neutral did not differ (*p* = 0.33), and Negative was lower than Neutral. Admiration and gratitude were also strongly positive, with small but reliable advantages of Ambivalent over Neutral/Negative and no discernible difference between Negative and Neutral (e.g., admiration: *p* = 0.74; gratitude: *p* = 0.17). Love mirrored this positive dominance, with a modest rise of Ambivalent over Neutral/Negative and a small elevation of Negative over Neutral. Full contrasts and adjusted p-values are reported in Supplementary Table S9.

##### Between-emotion Contrasts

**Fig. 2:**
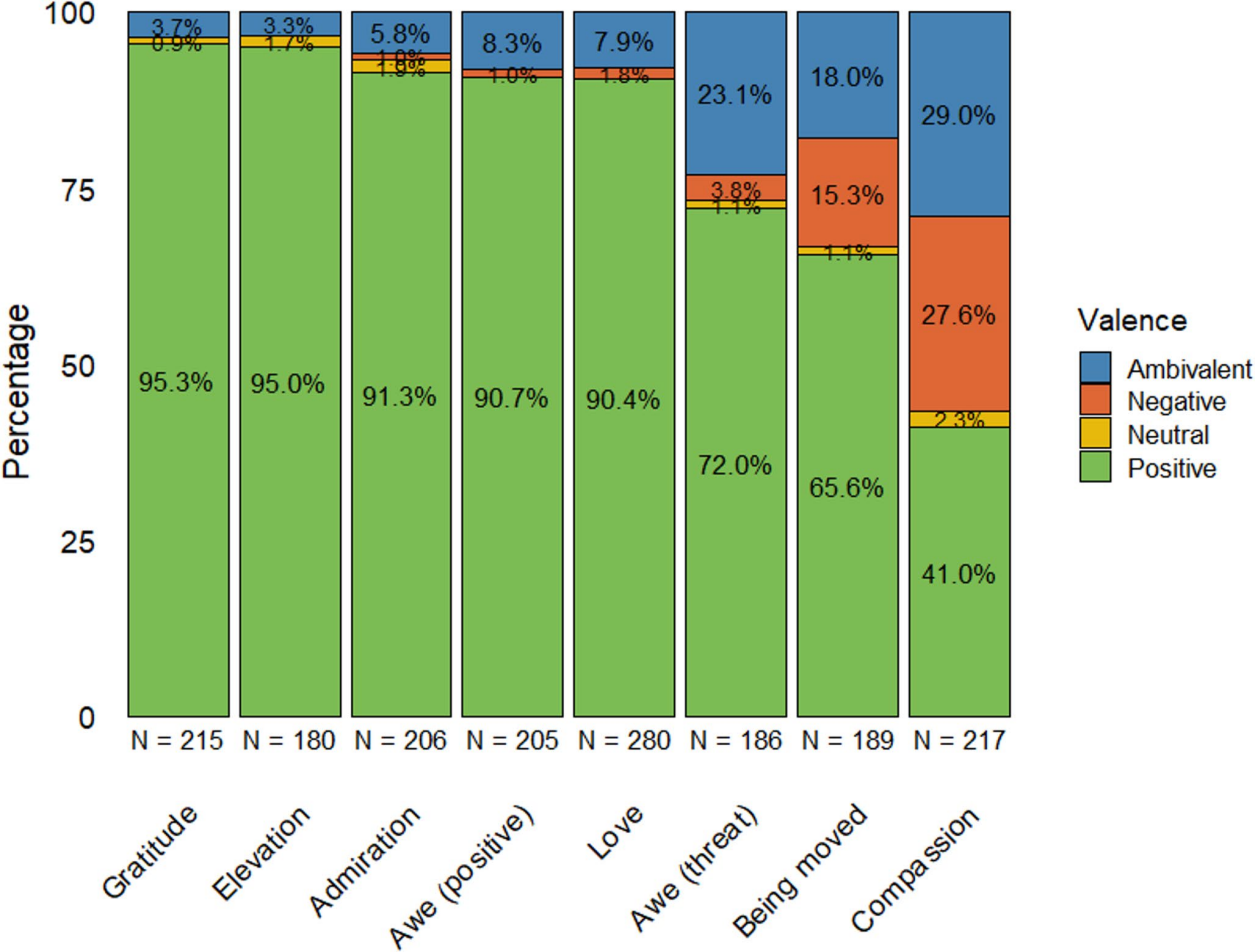
Distribution of emotional valence by emotion category. Stacked bar plots display the percentage of responses for each emotion category, subdivided into four valence levels (Positive, Negative, Ambivalent, Neutral). Each bar shows percentages of emotion experiences, and the total number of responses (N) for each emotion is annotated below the corresponding bar. Valence distributions are based on the combined coded dataset from Studies 2 and 3 and include only those who had experienced the emotion

For Positive appraisals, all emotions were generally high, but there was a clear gradient: gratitude showed the highest positive probability and was significantly higher than love; elevation, admiration, and positive awe were also very high and not reliably different from gratitude or love; threatening awe and commotion were significantly lower than these high-positive emotions; compassion was lowest. For Ambivalent appraisals, compassion had the highest probability and was significantly higher than commotion, threatening awe, positive awe, love, admiration, gratitude, and elevation (all pairwise differences significant), while threatening awe was elevated but not different from commotion. Positive awe and love formed a mid-low band, admiration and gratitude were lower, and elevation was lowest. For Negative appraisals, compassion was highest, significantly exceeding all other emotions; commotion was next highest and higher than elevation, gratitude, admiration, and positive awe; threatening awe and love were low (not reliably different from the near-zero levels of admiration and positive awe), and gratitude and elevation were almost zero. For Neutral appraisals, no reliable between-emotion differences emerged (Fig. 2). Full pairwise tests and adjusted p-values appear in Supplementary Table S10. 

## Study 4 – Clustering Emotion by Narrative Themes

### Method

#### Design and Participants

The design and participant pool for Study 4 were identical to those in Studies 2 and 3. The themes for the cluster analysis were drawn from the final coded dataset produced in those earlier studies.

### Data Analysis

To investigate how emotions might group based on narrative themes, we conducted a hierarchical cluster analysis using the pvclust package in R (Suzuki & Shimodaira, 2006). We constructed a data matrix in which each row represented a theme, and each column represented one of the investigated STEs. The entries reflected the frequency of each theme in each emotion category. Based on these frequencies, we calculated Euclidean distances among emotions and applied the complete linkage method to form clusters. To assess the robustness of the clusters, we computed selective inference (SI) *p*-values via multiscale bootstrap resampling (*N* = 10000), as described in Shimodaira and Terada (2019). This approach corrects for selection bias inherent in conventional hypothesis testing, which assumes that clusters are selected without prior knowledge of the data. By accounting for this bias, SI *p*-values offer a more accurate measure of cluster stability than approximately unbiased (AU) *p*-values. We considered SI values greater than 0.95 as evidence of a strongly supported cluster.

#### Theme–emotion Matrix, Missingness, and Sparsity Diagnostics

We aggregated narratives into an Emotion (rows) × Theme (columns) frequency matrix and converted each row to proportions. Zeros encode the absence of a theme within an emotion and do not indicate missing data; no imputation was applied. To reduce extreme sparsity, themes with ≤ 2 total occurrences were collapsed into “Others.” We report the matrix density (proportion of zeros), the average number of non-zero themes per emotion, and the narratives per emotion.

#### Tail-pruning Robustness Test

To assess whether tail sparsity could bias clustering, we re-ran pvclust (Euclidean distance; complete linkage; nboot = 10,000) while excluding “Others” and progressively pruning globally infrequent themes by increasing a minimum total-count cutoff from the rarest remaining theme (4 occurrences) up to the aggregate “Others” count (19 occurrences). For each cutoff, we rebuild the matrix (row-normalized) and refit pvclust, recording AU values. We summarize the Awe AU and whether any other cluster reached AU ≥ 0.95 across cutoffs.

### Results

Hierarchical clustering was performed on the emotion-by-theme matrix, and SI *p*-values were computed using multiscale bootstrap resampling (*N* = 10,000). Clusters with SI > 0.95 were deemed statistically robust. The final matrix exhibited a matrix density of 0.24, with emotions showing on average 22.8 non-zero themes (range 16 to 27). After collapsing ≤ 2-occurrence themes into “Others”, the rarest remaining theme was “Connection (social groups)” (4 occurrences), and the aggregate “Others” total had 19 occurrences.

**Fig. 3:**
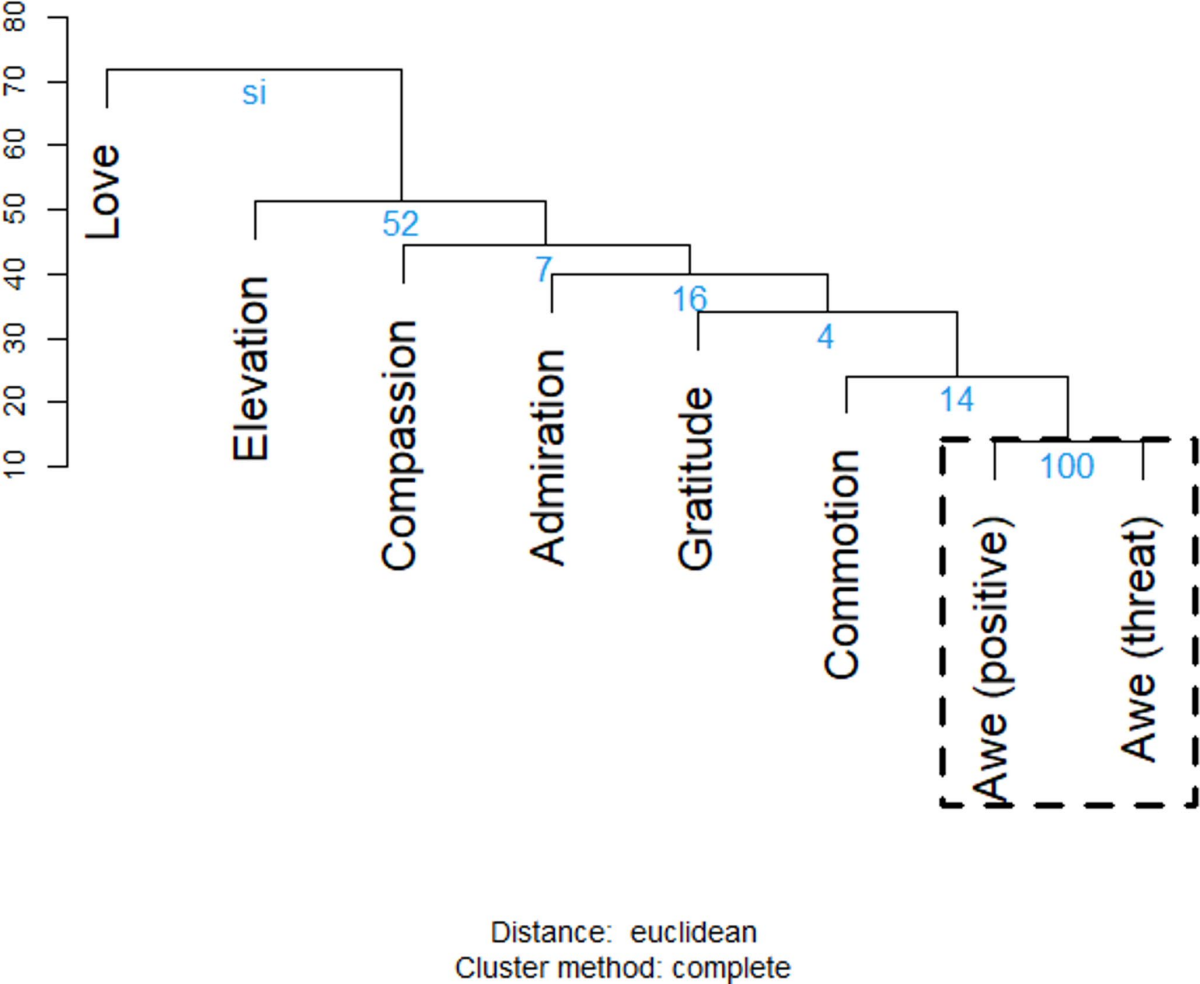
Clustering of emotions by themes. Hierarchical clustering of emotions based on Euclidean distances of themes and the complete linkage method. The dendrogram displays the relationships between emotions, with selective inference (SI) *p*-values shown in blue along the nodes. SI *p*-values were calculated via multiscale bootstrap resampling (*N* = 10,000). A dashed black rectangle outlines clusters with SI > 0.95, indicating statistically significant groupings

The analysis revealed one strongly supported cluster, grouping positive and threatening awe (SI = 1.00; see Fig. 3). In contrast, all other potential clusters exhibited low SI *p*-values, indicating the data did not strongly support their robustness or stability. Hence, only the positive–threat awe cluster emerged as statistically reliable, suggesting these two forms of awe share overlapping thematic content.

We performed a tail-pruning robustness test by increasing the global minimum total-count cutoff from 4 occurrences to the “Others” (*n* = 19 occurrences), which reduced the number of explicit theme columns from 28 to 23. Across all cutoffs, the Awe cluster’s AU ranged from 0.99 to 1, and no additional clusters achieved AU ≥ 0.95 (maximum AU = 0.90), indicating that the clustering is insensitive to tail sparsity.

**Fig. 4:**
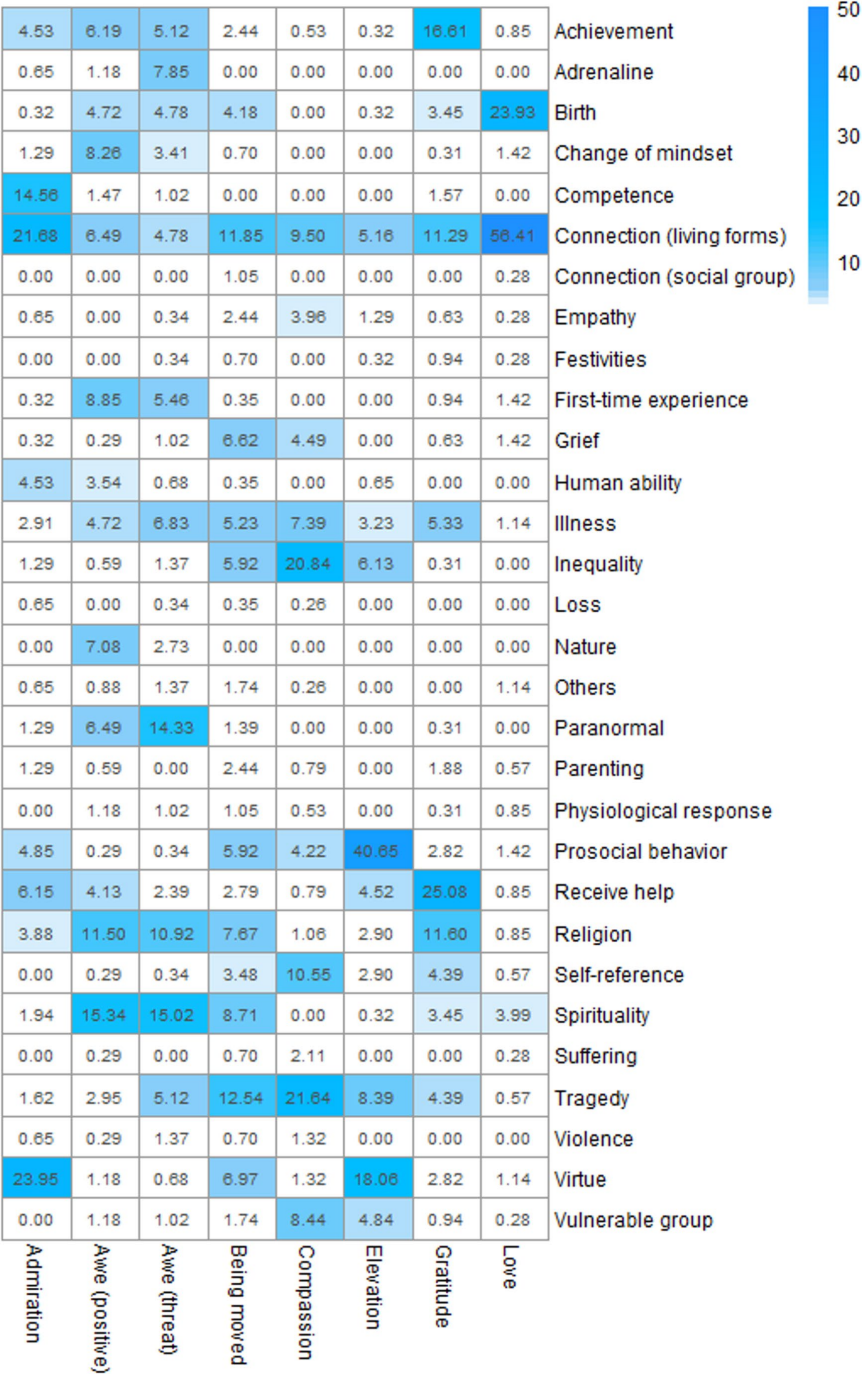
Distribution of themes across the emotional experiences. Heatmap illustrating the themes across the different emotions. Each cell represents the percentage of a given theme within an emotion. The color scale transitions from near-white (lower percentages) to dark blue (higher percentages), reflecting the relative prevalence of themes in each emotion

A heatmap (Fig. 4) illustrated the overlapping themes defining this cluster—namely, connection with living forms, achievement, birth, first-time experiences, paranormal phenomena, religion, and spirituality. These findings highlight that, despite notable differences in valence, both positive and threatening forms of awe share central thematic content. Although no other clusters were identified among the emotions, depicting the distribution of themes across emotions in the heatmap revealed that connection (living forms) appeared in narratives for all investigated emotions, albeit at lower frequencies for the emotions of threatening awe and elevation.

### General Discussion

Together, these studies shed new light on how STEs are experienced and structured in a culturally diverse Brazilian sample that diverges considerably from typical convenience samples.

Study 1’s primary contribution was the validation of open-ended items in Brazilian Portuguese STEs narratives. High values of comprehension among participants, both those who reported having the experience and those who did not, indicated that items were clear and understandable regardless of personal experience. Awe-related items required more revisions, yet their successful validation confirmed their effectiveness even without direct linguistic equivalents, underscoring that lacking specific emotion terms does not equate to the absence of emotional experiences (Fiske, 2020; Jackson et al., 2019). This indicates that emotional experiences can be culturally nuanced and deeply felt even when not explicitly named. Developing culturally sensitive assessment tools is essential to capture emotions that might otherwise remain overlooked due to linguistic differences. The validation procedure ensured that items were interpreted as intended, despite cross-language lexical gaps, by targeting the meanings derived from prior theoretical proposals. Thus, while language shapes how emotions are labeled and narrated, the successful validation of these items indicates access to the intended phenomenology without reliance on category names.

Study 2’s preliminary coding template identified 24 themes, expanded to 34 in Study 3. The most frequent themes – Connection (living beings), Tragedy, Prosocial Behavior, Virtue, and Receiving Help – accounted for 44% of all narratives, reflecting diverse STE experiences. These themes align well with theoretical expectations linking STEs to social connectedness, altruism, and moral motivations (Algoe & Haidt, 2009; Pizarro et al., 2021; Stellar et al., 2017; Yaden et al., 2017; Zahn et al., 2020).

The prevalence of tragedy themes likely reflects a regional disaster during data collection, illustrating how context impacts emotional narratives (Bonanno et al., 2010). Religion and Spirituality themes were also prominent, consistent with Brazil’s religious demographics (De Oliveira Torres, 1973; IBGE, 2013). This highlights how cultural context influences emotional experiences, particularly the integration of STEs with spiritual and religious sentiments (Emmons & Paloutzian, 2003; Yaden et al., 2017). Connection (living forms), the most recurring theme across emotions, highlights that STEs are relational and directed toward others or beyond the self. Yet, this shared core coexisted with substantial thematic diversity across emotions, indicating that STEs represent a heterogeneous constellation of experiences rather than a uniform emotional family.

Themes related to spirituality, religion, and the paranormal were prominent in both positive and threatening awe. These elements suggest that awe in Brazil is often interwoven with meaning systems that connect the individual to a divine or metaphysical order. This pattern diverges from findings in other cultures (e.g., Shiota et al., 2007; Stellar et al., 2024; Keltner, 2023), indicating that awe’s cognitive and affective structure flexibly incorporates culturally salient sources of vastness. Moreover, distinguishing between positive and threatening awe enabled a more nuanced analysis: while positive awe narratives involved novel experiences, threatening awe narratives emphasized adrenaline and tragedy. This empirical differentiation builds on Keltner and Haidt’s (2003) theoretical model by showing empirically that the qualities (or ‘flavors’) of awe are diverse and depend on contextual and cultural framing.

Compassion and being moved both centered on others’ suffering but differed in scope. Compassion emphasized empathic resonance with individuals, while Being moved narratives referenced collective distressing events, showing a more ambivalent/negative valence than previous studies (e.g., Cova & Deonna, 2014; Menninghaus et al., 2015). We attribute this to the context of data collection (e.g., flood news) but note that narratives still retained connection features, aligned within a social/self-transcendent emotion pattern rather than mere sadness.

The frequent Tragedy and Inequality themes in compassion, together with limited mentions of helping behavior, suggest that compassion narratives manifested affective attunement rather than explicit prosocial action, and fewer than half the participants reported positive compassion experiences. This challenges models that treat compassion as inherently prosocial and positive (Singer & Klimecki, 2014; Stellar et al., 2017) and aligns with constructivist perspectives emphasizing context and meaning construction over fixed motivational patterns (Condon & Feldman Barrett, 2013).

Aligned with previous research, elevation highlighted virtue and prosocial behavior amid adversity (Algoe & Haidt, 2009; Pohling & Diessner, 2016). Admiration’s themes overlapped with elevation in their emphasis on virtuous acts, but it also emphasized competence and achievement, highlighting admirable human qualities that motivate self-improvement (Algoe & Haidt, 2009; Schindler et al., 2013). Gratitude focused on receiving help and personal achievements (Wood et al., 2010), often with extended gratitude toward divine agents (Emmons & Crumpler, 2000). Love narratives focused on Connection (living forms) and the birth of a child, expressing enduring bonds with close others. This aligns with attachment-based accounts of love’s role in social cohesion (Feldman, 2017).

The theme-based clustering showed that only positive and threatening awe formed a statistically robust cluster, whereas the remaining STEs did not. This pattern underscores awe’s distinctiveness within the STE landscape, aligned with Abatista and Cova’s (2023) taxonomy, which separates awe and wonder into an Epistemic family (together with interest and surprise), rather than a Social family (i.e., emotions such as compassion, love, and being moved). Moreover, since no other thematic groupings emerged, this challenges the assumption that STEs form a tightly coherent emotional group. Yet, the shared connection theme highlights a unifying feature of STEs: their fundamental orientation beyond the self (Pizarro et al., 2021; Stellar et al., 2017).

The observed valence profiles across STEs indicate that these experiences are not uniformly appraised as positive. Gratitude, elevation, admiration, positive awe, and love were overwhelmingly classified as positive, whereas compassion and being moved showed more heterogeneous profiles, with substantial proportions of ambivalent and negative appraisals. Compassion displayed ambivalent or negative appraisals due to its inherent blend of warmth and distress, aligning with earlier findings (Goetz et al., 2010). Although most participants classified threatening awe as predominantly positive (72%), a notable proportion (23.1%) explicitly categorized it as ambivalent. Although this could be seen as in contradiction to previous work describing threat-awe as a mixed emotion (Chaudhury et al., 2022; Gordon et al., 2017; Nakayama et al., 2020; Stellar et al., 2024), the narratives frequently referenced adrenaline and overwhelming natural forces, indicating that threat-related components were present even when the overall appraisal was positive. This suggests that participants may retrospectively interpret these intense episodes more positively while still experiencing core ambivalent features. Methodologically, our use of a forced-choice categorical valence measure—unlike the continuous rating scales employed in previous studies—may have contributed to the lower proportion of ambivalent classifications.

### Limitations

Our sample was sampled from the general population, avoiding reliance on student samples. Yet, the cultural and religious contexts specific to Brazil may limit the applicability of our findings to other regions. A constructivist perspective suggests caution in extending our context-bound findings to other cultural settings (Mesquita et al., 2016). For example, themes such as religious awe, prominent in our Brazilian sample, may not appear with the same salience in less religious contexts, yet they demonstrate how cultural context influences the experience of STEs. However, rather than broadly generalizing specific themes, our dataset illustrates general principles of how context shapes emotional expression. These situated insights thus inform broader emotion models by highlighting the need to incorporate cultural and situational factors. Moreover, our online data collection method inherently excludes individuals lacking internet access or with lower literacy levels, underrepresenting lower socioeconomic and educational strata. Relying on participants’ self-reported narratives of their most impactful emotional experiences introduces a bias toward memorable rather than everyday experiences. While narratives richly described situational contexts and elicitors, we were unable to consistently code appraisal dimensions (e.g., certainty, control, responsibility), which we flag as a valuable direction for future work.

## Conclusion

Our data provides partial support for both universalist and constructivist accounts. Successful validation of emotion-specific items suggests that certain core emotional components may indeed be universal, allowing consistent recognition across cultures. This universalist account is strengthened by the emergence of recognizable awe experiences in narratives, because a specific term is lacking in Portuguese. Moreover, participants frequently described STEs’ experiences with components expected by theory, and the most ubiquitous theme across all eight emotions was Connection with living forms, indicating that a relational focus is indeed a common core feature of STEs in our sample. These convergences on predicted core appraisals and relational themes suggest a degree of universality or an “essence” in these experiences.

At the same time, our findings of specific themes diverge in important ways from observations in other cultural contexts, underscoring a constructionist view. For instance, experiences coded under Religion/Spirituality figured prominently among the top themes in our Brazilian sample—perhaps reflecting Brazil’s cultural milieu of high religious engagement—whereas in less religious populations or with different religious traditions, secular contexts (such as awe in nature or personal accomplishment) tend to be more dominant (e.g., Shiota et al., 2007).

Our results also contribute to discussions about whether STEs form a distinct emotional category. Although STEs share an outward-directed focus, their thematic diversity, cultural variability, and complex valence profiles suggest they are neither completely homogeneous nor entirely unrelated. Instead, STEs constitute a spectrum united by a sense of connection to something beyond oneself, manifested across diverse situations. Thus, our findings underscore the value of nuanced, integrative models that acknowledge both shared psychophysiological processes and culturally situated meanings potentially bridging basic emotion and constructivist theories (Friedman & Thayer, 2024).

## Supplementary Information

Below is the link to the electronic supplementary material.

Supplementary Material 1

## Additional Information

### Availability of Data and Material

All studies’ materials, data, and analysis scripts are publicly available at https://osf.io/7r6n4.
