# Supplementary material for: Mapping the Content Landscape of Self-transcendent Emotional Experiences with Thematic Analysis and Hierarchical Clustering
Source: Affect Sci. 2026 Mar 19;7(2):232–52. doi: 10.1007/s42761-026-00356-x (PMC13269614; doi:10.1007/s42761-026-00356-x)
Supplement: Supplementary file 1 — Supplementary Material 1 [file 42761_2026_356_MOESM1_ESM.docx]

**Supplementary materials**

**Mapping the content landscape of self-transcendent emotional experiences using a mixed-method approach**

**Study 1**

**Supplementary Methods**

Table S1. Sample description of item validation study (Study 1)

| **Variable** | **N = 362**^1^ |
| --- | --- |
| **Social class** |  |
| A | 66 (19%) |
| B1 | 63 (18%) |
| B2 | 110 (32%) |
| C1 | 56 (16%) |
| C2 | 37 (11%) |
| D-E | 13 (3.8%) |
| Unknown | 17 |
| **Education** |  |
| Up to incomplete secondary school | 14 (4.1%) |
| Undergraduate degree incomplete | 151 (44%) |
| Undergraduate degree completed | 106 (31%) |
| Postgraduate degree | 74 (21%) |
| Unknown | 17 |
| **Age** |  |
| Mean (+-SD) | 43.1 (+-12.78) |
| Range | 18.0, 72.00 |
| Unknown | 17 |
| **Regions** |  |
| Center-West | 32 (9.3%) |
| North | 20 (5.8%) |
| Northeast | 64 (19%) |
| South | 55 (16%) |
| Southeast | 174 (50%) |
| Unknown | 17 |
| **Sex** |  |
| Female | 175 (51%) |
| Male | 170 (49%) |
| Unknown | 17 |
| ^1^n (%) | |

**Supplementary Results**

**Table S2.** Final item wording for self-transcendent emotions in Portuguese and their direct English translation.

| **Target emotion (Portuguese translation)** | **Item Wording Portuguese** | **Item Wording English direct translation** |
| --- | --- | --- |
| Admiration  (Admiração) | Eu tive uma experiência na qual senti muita admiração por uma pessoa que se destacou de todas as outras experiências do tipo que eu já tive. | I had an experience in which I felt a great deal of admiration for a person who stood out from all other similar experiences I have had. |
| Awe (positive)*  (Deslumbramento; Fascínio; Maravilhamento) | Eu tive uma experiência em que fiquei fascinado e maravilhado ao ver, sentir ou perceber algo grandioso de uma forma nova que desafiou como eu pensava até então. | I had an experience in which I was fascinated and amazed to see, feel, or perceive something grand in a new way that challenged my previous thoughts. |
| Awe (threatening)  (Deslumbramento; Fascínio; Maravilhamento) | Eu tive uma experiência que, apesar de ser assustadora, me gerou profundo maravilhamento e fascínio. | I had an experience that, despite being frightening, filled me with profound wonder and fascination. |
| Being moved (Comoção) | Eu tive uma experiência em que me senti muito tocado e comovido que se destacou de todas as experiências do tipo que já tive. | I had an experience that deeply moved and touched me, standing out from all similar experiences I have had. |
| Compassion*  (Compaixão) | Eu tive uma experiência específica em que senti compaixão pelo sofrimento de outros (humanos ou não) que se destacou de todas as outras experiências do tipo que eu ja tive. | I had a specific experience in which I felt compassion for the suffering of others (human or non-human), which stood out from all other similar experiences I have had. |
| Elevation  (Elevação) | Eu tive uma experiência em que vi alguém realizar um grande ato de bondade ou caridade para outra pessoa. | I had an experience in which I witnessed someone perform a great act of kindness or charity towards another person. |
| Gratitude  (Gratidão) | Eu tive uma experiência na qual senti muita gratidão e que se destacou de todas as experiências do tipo que já tive. | I had an experience of immense gratitude, which stands out from all the others I have had. |
| Love*  (Amor) | Eu já senti amor de uma forma que se destacou de todas as outras experiências do tipo que eu já tive. | I have had an experience of love that stands out from all others. |

*Note:* * Items validated as part of the Brazilian validation of the original Inventory of Non-Ordinary Experiences (INOE-BR; Fischer et al. 2025).

**Table S3.** *Summary of validation results for all versions of each item, validated and non-validated.*

| **Item** | **Iteration** | **N†** | **ExpU** | **ExpNU** | **3s** | **VS** | **PPU** | **NPU** | **IDK** | **Wording** | **Example narrative** |
| --- | --- | --- | --- | --- | --- | --- | --- | --- | --- | --- | --- |
| Admiration | 1 | 56 | 46 | 5 | 5 | 90.2 | 90.9 | 88.9 | 7 | I had an experience in which I felt a great deal of admiration for a person who stood out from all other similar experiences I have had. | "I have a friend who went through a very big trauma (she lost a daughter at the age of five), and since then, she has become a Buddhist and a midwife. She is very inspiring, and when she speaks, I absorb all of her strength, spirituality, resilience, and altruism." |
| Awe (positive) | 1 | 40 | 23 | 8 | 9 | 74.2 | 78.3 | 62.5 | 5 | I had an experience of fascination or wonder that stood out from all the other experiences of this kind I have ever had. | “I was fascinated when I was able to choose my house.”; “The presentation and conclusion of my final thesis project at the technical school.” |
| Awe (positive) | 2 | 42 | 24 | 2 | 16 | 92.3 | 94.1 | 88.9 | 6 | I had an experience in which I was fascinated and amazed to see, feel, or perceive something grand in a new way that challenged my previous thoughts. | "When I first saw the Iguaçu Falls, I was fascinated by the grandeur of nature and by God. I never imagined something so powerful and majestic could exist." |
| Awe (threat) | 1 | 10 | 0 | 2 | 8 | 0.0 |  | 0.0 | 2 | I had an experience of astonishment that left me perplexed and stood out from all the other such experiences I have ever had. | “I went to a client’s house and she treated me really badly, even though I had already sold to this client several times.”; “Making a simple comment and seeing it spiral out of control against me.” |
| Awe (threat)* | 2 | 34 | 20 | 5 | 9 | 80.0 | 76.9 | 83.3 | 6 | I had an experience that, despite being frightening, filled me with profound wonder and fascination. | "A boat ride in Bonito, MS. I don’t know how to swim, so I was terrified, but at the same time, I was fascinated by the beauty of the place and the adrenaline I felt. It was truly incredible. Fear and wonder at the same time. I’ve never forgotten it." |
| Awe (threat) | 3 | 33 | 13 | 12 | 8 | 52.0 | 47.4 | 66.7 | 2 | I had an experience of deep fascination or amazement that, at the same time, generated anxiety or fear | “I was robbed.”; “I felt anxious and very scared during the pandemic. I was afraid of returning to my routine. I was afraid of dying.” |
| Awe (threat) | 4 | 12 | 2 | 3 | 7 | 40.0 | 50.0 | 33.3 | 1 | I had an experience that, although frightening and causing me fear, also left me fascinated because it was something grand that went beyond anything I had ever experienced before. | “The situation of having left my home state and moved to another one I didn’t even know. After years of struggling and trying to improve my life, I managed to pass a public service exam, and today I am married, have a child, and live well.” |
| Awe (threat) | 5 | 29 | 17 | 8 | 4 | 68.0 | 60.0 | 80.0 | 8 | I once had an experience in which I felt fascinated or amazed by something, while at the same time feeling dread or fright. | “I met someone who asked me out, but I was afraid of getting involved. I knew he wanted something serious, and I wasn’t sure if that was what I wanted.” |
| Being moved | 1 | 55 | 44 | 1 | 10 | 97.8 | 97.5 | 100.0 | 3 | I had an experience that I felt deeply moved and touched, standing out from all similar experiences I have had. | "When I saw my father in a sort of dream on the night he passed away. He just looked at me with the same tender gaze he always had." |
| Compassion | 1 | 52 | 47 | 1 | 4 | 97.9 | 97.6 | 100.0 | 4 | I had a specific experience where I felt compassion for the suffering of others (human or non-human), standing out from all other similar experiences I have had. | “A family stuck in their home due to heavy rain and a landslide had to be rescued by neighbors.” |
| Elevation | 1 | 56 | 41 | 2 | 13 | 95.3 | 100.0 | 71.4 | 1 | I had an experience where I witnessed someone perform a great act of kindness or charity towards another person. | “A guy from my workplace gave a basic food kit to a lady and her son who were asking for help.” |
| Gratitude | 1 | 49 | 42 | 3 | 4 | 93.3 | 92.3 | 100.0 | 8 | I had an experience in which I felt immense gratitude, standing out from all similar experiences I have had. | “My father fell ill at home, and someone helped us get to the hospital, stayed with me there, and brought us back home. I’m very grateful for that.” |
| Love | 1 | 53 | 39 | 4 | 10 | 90.7 | 100.0 | 60.0 | 6 | I have had an experience of love that stood out from all other such experiences. | “When my daughters were born.”; “When I prayed and opened my heart to God, I could feel His presence with me, as well as His peace and confort.” |

**Note:** N Exp = number of participants who reported having experienced the specific emotion but not necessarily demonstrating a correct understanding of the item; Exp U = number of participants who reported having experienced the particular emotion and demonstrated a correct understanding of the corresponding item; VS = Validation Score; PPU = positive proportion understood; The total N contains all valid responses per item in that iteration, 3 valued answers are not accounted for VS calculation. * The validated version was the one from iteration 2. However, due to the VS matching the cut-off, we tried other versions that did not pass.

**Study 2 – Initial coding template of STE Narratives**

**Supplementary Methods**

**Supplementary Table S4.** Sociodemographic descriptives in samples from Studies 2 and 3.

| **Sample** | **Study 2**  N = 324 | **Study 3**  N = 453^1^ |
| --- | --- | --- |
| **Age** | 45 (+-14) | 43 (+-13) |
| **Sex** |  |  |
| Male | 186 (57%) | 185 (41%) |
| Female | 138 (43%) | 268 (59%) |
| **Region** |  |  |
| North | 24 (7.4%) | 30 (6.6%) |
| Northeast | 77 (24%) | 102 (23%) |
| Southeast | 123 (38%) | 181 (40%) |
| South | 75 (23%) | 81 (18%) |
| Center-West | 25 (7.7%) | 59 (13%) |
| **Social class** |  |  |
| A | 80 (25%) | 85 (19%) |
| B1 | 70 (22%) | 99 (22%) |
| B2 | 78 (24%) | 81 (18%) |
| C1 | 60 (19%) | 66 (15%) |
| C2 | 22 (6.8%) | 53 (12%) |
| D-E | 14 (4.3%) | 69 (15%) |
| **Education** |  |  |
| Up to incomplete secondary school | 22 (6.9%) | 38 (8.5%) |
| Undergraduate degree incomplete | 112 (35%) | 200 (45%) |
| Undergraduate degree completed | 101 (32%) | 110 (25%) |
| Postgraduate degree | 83 (26%) | 100 (22%) |
| Unknown | 6 | 5 |
| **Religion affiliation^2^** |  |  |
| No religion | - | 78 (18%) |
| Catholic | - | 166 (38%) |
| Protestant | - | 38 (8.6%) |
| Pentecostal evangelical | - | 64 (15%) |
| Neo-Pentecostal Evangelical | - | 10 (2.3%) |
| Jewish | - | 1 (0.2%) |
| Buddhist | - | 3 (0.7%) |
| Spiritist | - | 36 (8.2%) |
| Afro-Brazilian | - | 22 (5.0%) |
| Other | - | 23 (5.2%) |
| Unknown | 337 | 11 |
| ^1^Mean (+-SD); n (%)  ^2^Religion affiliation was asked only in Study 3 | | |

**Supplementary Results**

**Supplementary Table S5.**

*Themes identified in the initial coding template of study 1 narratives. The final template with theme descriptions was created, taking into account the entire dataset of Studies 2 and 3, and is shown in Table 2.*

| **Theme** | **% Occurrence** | **Cumulative %** |
| --- | --- | --- |
| Love | 11.01% | 11.01% |
| Emotional bond | 7.72% | 18.73% |
| Prosocial behavior | 7.09% | 25.82% |
| Virtue | 6.67% | 32.49% |
| Inequality | 5.71% | 38.20% |
| Religion | 5.61% | 43.81% |
| Spirituality | 5.40% | 49.21% |
| Tragedy | 4.87% | 54.07% |
| Birth | 4.76% | 58.84% |
| Receive help | 4.66% | 63.49% |
| Achievement | 4.23% | 67.72% |
| Unprecedented | 4.23% | 71.96% |
| Self-reference | 3.39% | 75.34% |
| Specific groups | 3.17% | 78.52% |
| Gratitude | 3.07% | 81.59% |
| Illness | 2.96% | 84.55% |
| Sensitivity | 2.65% | 87.20% |
| Competence | 2.54% | 89.74% |
| Nature | 2.12% | 91.85% |
| Grief | 2.01% | 93.86% |
| NOE | 2.01% | 95.87% |
| Parenting | 1.80% | 97.67% |
| Human ability | 1.27% | 98.94% |
| Adrenaline | 1.06% | 100.00% |

**Study 3 – Refining the template coding**

**Supplementary Methods**

**Supplementary Table S6.**

*Complete crosswalk of changes (Preliminary theme → Final theme) after refinement of the coding template from Study 2 to 3.*

| **Preliminary themes** | **Final themes** | **n** | **prop_of_changes** |
| --- | --- | --- | --- |
| Love | Connection (living forms) | 200 | 25.91 |
| Emotional bond | Connection (living forms) | 179 | 23.19 |
| NOE | Paranormal | 58 | 7.51 |
| Specific groups | Vulnerable group | 56 | 7.25 |
| Unprecedented | First-time experience | 54 | 6.99 |
| Unprecedented | Change of mindset | 46 | 5.96 |
| Sensitivity | Empathy | 31 | 4.02 |
| Gratitude | Religion | 24 | 3.11 |
| Connection | Connection (living forms) | 13 | 1.68 |
| Gratitude | Connection (living forms) | 13 | 1.68 |
| Love | Spirituality | 13 | 1.68 |
| Connection | Spirituality | 11 | 1.42 |
| Sensitivity | Virtue | 9 | 1.17 |
| Gratitude | Self-reference | 8 | 1.04 |
| Gratitude | Achievement | 5 | 0.65 |
| Gratitude | Prosocial behavior | 4 | 0.52 |
| Sensitivity | Prosocial behavior | 4 | 0.52 |
| Gratitude | Illness | 3 | 0.39 |
| Gratitude | Parenting | 3 | 0.39 |
| Gratitude | Spirituality | 3 | 0.39 |
| Love | Connection (social group) | 3 | 0.39 |
| Beauty | Others | 2 | 0.26 |
| Gratitude | Tragedy | 2 | 0.26 |
| Love | Self-reference | 2 | 0.26 |
| Nostalgia | Others | 2 | 0.26 |
| Other | Others | 2 | 0.26 |
| Sacrifice | Others | 2 | 0.26 |
| Shame | Others | 2 | 0.26 |
| Connection | Connection (social group) | 1 | 0.13 |
| Death | Loss | 1 | 0.13 |
| Disappointment | Others | 1 | 0.13 |
| Gratitude | Empathy | 1 | 0.13 |
| Gratitude | Festivities | 1 | 0.13 |
| Gratitude | Inequality | 1 | 0.13 |
| Gratitude | Loss | 1 | 0.13 |
| Gratitude | Others | 1 | 0.13 |
| Gratitude | Suffering | 1 | 0.13 |
| Love | Illness | 1 | 0.13 |
| Love | Others | 1 | 0.13 |
| Love | Parenting | 1 | 0.13 |
| Love | Prosocial behavior | 1 | 0.13 |
| Sensitivity | Others | 1 | 0.13 |
| Sensitivity | Receive help | 1 | 0.13 |
| Spirituality | Paranormal | 1 | 0.13 |
| Spirituality | Religion | 1 | 0.13 |
| Unprecedented | Others | 1 | 0.13 |

**Supplementary Results**

**Supplementary Table S7.**

*Most frequent themes and representative narratives by emotion. For each emotion, the most frequent themes (accounting for approximately 80% of total theme occurrences within the emotional category) are shown, along with one representative self-report excerpt illustrating each theme.* *Only English direct translations of the excerpts are presented here; the original Portuguese data are available in the OSF repository.*

| **Emotion** | **Theme** | **Example narrative^*^** |
| --- | --- | --- |
| Admiration | Virtue | “The way the doctor treated a patient, without any financial gain, purely out of love for the profession.” |
|  | Connection (living forms) | “When my son was born, my wife had difficulties, mainly with postpartum depression. The way the experience of being a mother helped her overcome all of this made me admire her even more.” |
|  | Competence | “When a co-worker managed to accomplish a task that everyone said was impossible.” |
|  | Receive help | “It was at the technical school where I was studying in high school; the nursing coordinator at the school donated a complete white uniform from nursing to me, and I was able to continue my studies there.” |
|  | Prosocial behavior | “When I witness situations in which a person gives from the little they have to help another. My mother did this her whole life.” |
|  | Human ability | “An experience at an international concert, where I was able to see the talent of a great idol up close.” |
|  | Achievement | “When I saw my girlfriend releasing a book.” |
| Awe (positive) | Spirituality | “I went through a traffic accident, almost to the point of death, but a higher power brought me back.” |
|  | Religion | “It was a spiritual experience… I had recently lost my father without being able to say goodbye. In the midst of depression, I went to the Spiritist center, where they advised me to undergo disobsession. During one session, a medium who was accompanying us received an entity that embraced me and said it only wanted to say goodbye… it was emotional!” |
|  | First-time experience | “I had the opportunity to fly over the Swiss Alps, and it was when I saw snow for the first time. The light of the sunrise over those mountains and the glow of the snowy peaks made me realize how grand nature is, and how we are almost insignificant, even while being thousands of meters above it.” |
|  | Change of mindset | “I passed an exam that I thought I would never be able to because of its level of complexity. That made me believe in myself more.” |
|  | Nature | “It was on a trip with my family. We were walking on a pier on the coast of São Paulo and came across a mind-blowing sight. Near the pier there was a humpback whale with her calf. We spent hours admiring that sight. It was an unforgettable moment for all of us!” |
|  | Paranormal | “I lived on a small farm and spotted in the sky circular lights that formed two large circles, they came closer and suddenly it flew at a speed unlike anything I'd seen and vanished on the horizon. Then I realized there are many things beyond here. I saw it and so did some neighbors, I found it fascinating.” |
|  | Connection (living forms) | “The first time I looked at the face of a young man, who flatly refused to look at me, I felt that I fell in love at first sight with him. And I thought in that same instant: I want him to be the father of my child.” |
|  | Achievement | “Visiting a magical place that I had dreamed of for many years, which is the Serra Gaúcha and its charms.” |
|  | Illness | “When I was transplanted.” |
|  | Birth | “I was present for my wife's labor and the birth of my children.” |
| Awe (threat) | Spirituality | “I had a spiritual experience where I was touched by someone who was not there.” |
|  | Paranormal | “When I saw a spirit with a staff in its hand, but the face was as if it were one of those store-window mannequins... It had a shape but had no eyes, nose, or mouth.” |
|  | Religion | “It was at an Umbanda terreiro, where I had contact with spirituality, and at the same time that it was scary, it was a very good feeling.” |
|  | Adrenaline | “I climbed a rock with my own hands on the Brazil–Bolivia border.” |
|  | Illness | “My sister suffered a stroke at 7 years of age; she even passed away in my father's arms, who prayed and asked God for her to come back to life; she came back to life and went to the hospital, spent some time in a coma (...); the doctors said she would have sequelae and would live until 13; she had no sequelae, she is now 33 years old (...)” |
|  | First-time experience | “I participated in an Umbanda service, and I saw for the first time someone receive an entity.” |
|  | Tragedy | “The water arriving in my city were frightening, but at the same time it caused me a sense of wonder even though the lagoon was quite full, the landscape was beautiful.” |
|  | Achievement | “When participating in the samba schools parade of my state's Carnival for the first time as a front-commission dancer (the school's first section to enter the avenue), I was very frightened by the number of people, cameras, judges, but it was wonderful.” |
|  | Connection (living forms) | “My dog was very sick and with no prospect of improvement after surgery to remove a tumor from his spleen. Due to his intense suffering, we decided to euthanize him and, with great pain in our hearts, we said goodbye to our beloved pet. A week later, after a period of crying and pain in the soul, the veterinarian got in touch saying that our little one was alive and slowly recovering. We were astonished at his fight for life. Even with so much pain, this situation made me value life, family, and all the people I care about.” |
|  | Birth | “I had this experience when I found out my girlfriend was pregnant; it was frightening to have a child and have to take care of him, but at the same time wonderful, because I really wanted to have a child!” |
| Being moved | Tragedy | “Interviews with people who lost everything in the floods in Rio Grande do Sul.” |
|  | Connection (living forms) | “A very close cousin passed away from COVID during the pandemic. I spoke with her on the eve of her death... I was very moved thinking about her children.” |
|  | Spirituality | “I was receiving a prayer from a person. I began to feel something good passing through my body.” |
|  | Religion | “When I was a teenager at the school where I studied, they showed a video about Jesus, and that story moved me a lot.” |
|  | Virtue | “When I witness the kindness of people. The suffering and the faith.” |
|  | Grief | “The death of a patient whom I treated for months.” |
|  | Prosocial behavior | “I am always moved when I see a life being saved from death or abandonment, whether human or animal, because for me every life is important.” |
|  | Inequality | “Near Christmas, a couple came into my store asking to clean the display window because they needed money to eat. The woman was pregnant, almost about to have the baby; the couple was extremely humble.” |
|  | Illness | “When my grandfather was alive and ill and I was a child and I found myself doing physical therapy on him without knowing what physical therapy was, but trying somehow to help, that marked me deeply and over time I understood what I was doing.” |
|  | Birth | “The birth of my younger brother. When I could see him and hold him for the first time and feel an extraordinary love for him.” |
|  | Self-reference | “A feeling of great sadness I felt about what happened in Rio Grande do Sul, with people having only the clothes on their backs, when we put ourselves in the person’s place it is despairing.” |
| Compassion | Tragedy | “All the images of wars or disasters. When I see the situation of the general population suffering from the lack of basic necessities.” |
|  | Inequality | “When I see people sleeping on the sidewalks, it makes me very sad, for a human being to live in such a difficult situation, without even the basics to live with a bit of dignity.” |
|  | Self-reference | “When I saw someone receive the call about her mother's passing right in front of me, even though I had no relation to her I felt her pain. Because it could have been my mother, I put myself in her place.” |
|  | Connection (living forms) | “When my brother was immersed in drugs and hanging out with the wrong people, I felt that I should help him and not judge him.” |
|  | Vulnerable group | “A little girl was suffering at the hands of her parents, and I was very moved by her situation, and to this day I think about how she is at this moment.” |
|  | Illness | “When I saw my aunt with cancer wasting away, because of the difficulty eating and for being so thin.” |
| Elevation | Prosocial behavior | “I saw a news report where a motorcyclist covered a homeless person with a blanket.” |
|  | Virtue | “The true story of Sister Dulce, the good angel of Bahia.” |
|  | Tragedy | “When many Brazilians from all states came together to help the victims of Rio Grande do Sul, which suffered a tragedy.” |
|  | Inequality | “I saw a father counting coins to buy food for his children, and in the end he couldn't pay. A lady came by, witnessed everything, and offered to pay for the groceries.” |
|  | Connection (living forms) | “A coworker at the company who lived alone fell ill, was put on leave, and another coworker from the same company went out of her way to help her, going to her house every day; and when she couldn’t, her husband went there to bring food and see how the coworker was doing.” |
| Gratitude | Receive help | “When I was going through emotional and financial problems, I was supported all the time by my wife; she believed in me even when others had already given up.” |
|  | Achievement | “Gratitude for having started to work after 10 years.” |
|  | Religion | “The sacrifice of Jesus on the Cross for my life and for all humanity.” |
|  | Connection (living forms) | “I am very grateful to my uncle for always being by my side.” |
|  | Illness | “When my friends sought outside help when I had suicidal tendencies. It was a moment of very strong closeness, and to this day I am grateful to them for having done that.” |
|  | Tragedy | “I needed help to identify my deceased mother's body because I had COVID. Because the person did for me what I should have done.” |
|  | Self-reference | “I felt deep gratitude while doing a meditation in a Zen Buddhist space, where I remembered how good it is to have health, family, a home to live in, etc.” |
| Love | Connection (living forms) | “I met someone with whom I felt a huge connection; we had a relationship, and my feelings for him were stronger than in any other relationships.” |
|  | Birth | “I felt love for my daughter at her birth; I saw that it was such a pure love that it almost hurt from so much love.” |

*Note: * literal translation performed by LLM GPT-5 Thinking. Original narratives in Portuguese can be seen in the dataset at the project repository*

*Self-Reported Valence of Experiences*

*Table S8, Valence probabilities by emotion (model-based)*

| **Emotion** | **Positive** | **Ambivalent** | **Negative** | **Neutral** |
| --- | --- | --- | --- | --- |
| Admiration | 91.6 [88.3–94.8] | 5.5 [2.8–8.2] | 1.3 [-0.0–2.6] | 1.6 [0.1–3.1] |
| Awe (positive) | 91.4 [88.3–94.6] | 7.4 [4.4–10.3] | 1.2 [-0.0–2.4] | 0.0 [-0.0–0.0] |
| Awe (threat) | 71.0 [65.5–76.5] | 24.2 [19.1–29.4] | 3.8 [1.5–6.0] | 1.0 [-0.2–2.2] |
| Commotion | 65.9 [60.1–71.6] | 18.5 [13.7–23.2] | 15.0 [10.6–19.3] | 0.7 [-0.3–1.7] |
| Compassion | 42.0 [36.7–47.2] | 30.6 [25.7–35.5] | 25.3 [20.7–29.9] | 2.1 [0.6–3.6] |
| Elevation | 94.8 [92.2–97.4] | 3.2 [1.2–5.3] | 0.0 [-0.0–0.0] | 1.9 [0.3–3.6] |
| Gratitude | 95.3 [92.8–97.7] | 4.1 [1.8–6.4] | 0.0 [-0.0–0.0] | 0.6 [-0.3–1.6] |
| Love | 90.6 [87.4–93.8] | 7.7 [4.8–10.6] | 1.7 [0.3–3.1] | 0.0 [0.0–0.0] |
| Notes. Entries are model-based probabilities (%) with 95% Wald CIs, estimated from a multinomial logistic regression (nnet::multinom) and marginal means on the probability scale (emmeans, type = 'response'). | | | | |

Table S9. Within-emotion contrasts of valence probabilities.

| **Emotion** | **Contrast** | **Difference (pp) [95% CI]** | **Adj. p** |
| --- | --- | --- | --- |
| Admiration | Positive - Ambivalent | 86.1 [78.2–93.9] | p < .05 |
|  | Positive - Negative | 90.3 [84.8–95.8] | p < .05 |
|  | Positive - Neutral | 90.0 [84.2–95.7] | p < .05 |
|  | Ambivalent - Negative | 4.2 [-0.0–8.4] | p < .05 |
|  | Ambivalent - Neutral | 3.9 [-0.4–8.2] | p < .05 |
|  | Negative - Neutral | -0.3 [-3.1–2.5] | p = 0.7425 |
| Awe (positive) | Positive - Ambivalent | 84.1 [75.8–92.4] | p < .05 |
|  | Positive - Negative | 90.3 [85.1–95.5] | p < .05 |
|  | Positive - Neutral | 91.4 [87.1–95.8] | p < .05 |
|  | Ambivalent - Negative | 6.2 [1.7–10.7] | p < .05 |
|  | Ambivalent - Neutral | 7.4 [3.3–11.5] | p < .05 |
|  | Negative - Neutral | 1.2 [-0.5–2.9] | p = 0.0556 |
| Awe (threat) | Positive - Ambivalent | 46.8 [32.4–61.1] | p < .05 |
|  | Positive - Negative | 67.2 [58.1–76.4] | p < .05 |
|  | Positive - Neutral | 70.0 [61.9–78.0] | p < .05 |
|  | Ambivalent - Negative | 20.5 [12.3–28.7] | p < .05 |
|  | Ambivalent - Neutral | 23.2 [15.7–30.7] | p < .05 |
|  | Negative - Neutral | 2.7 [-0.9–6.4] | p < .05 |
| Commotion | Positive - Ambivalent | 47.4 [34.0–60.7] | p < .05 |
|  | Positive - Negative | 50.9 [38.3–63.5] | p < .05 |
|  | Positive - Neutral | 65.2 [56.8–73.5] | p < .05 |
|  | Ambivalent - Negative | 3.5 [-6.3–13.3] | p = 0.3168 |
|  | Ambivalent - Neutral | 17.8 [11.0–24.6] | p < .05 |
|  | Negative - Neutral | 14.3 [8.0–20.6] | p < .05 |
| Compassion | Positive - Ambivalent | 11.3 [-1.1–23.8] | p < .05 |
|  | Positive - Negative | 16.6 [4.8–28.5] | p < .05 |
|  | Positive - Neutral | 39.8 [32.0–47.7] | p < .05 |
|  | Ambivalent - Negative | 5.3 [-5.7–16.3] | p = 0.1812 |
|  | Ambivalent - Neutral | 28.5 [21.2–35.8] | p < .05 |
|  | Negative - Neutral | 23.2 [16.3–30.2] | p < .05 |
| Elevation | Positive - Ambivalent | 91.6 [85.5–97.8] | p < .05 |
|  | Positive - Negative | 94.8 [91.2–98.5] | p < .05 |
|  | Positive - Neutral | 92.9 [87.6–98.2] | p < .05 |
|  | Ambivalent - Negative | 3.2 [0.3–6.1] | p < .05 |
|  | Ambivalent - Neutral | 1.3 [-2.4–5.0] | p = 0.3266 |
|  | Negative - Neutral | -1.9 [-4.2–0.3] | p < .05 |
| Gratitude | Positive - Ambivalent | 91.1 [84.6–97.7] | p < .05 |
|  | Positive - Negative | 95.3 [91.8–98.7] | p < .05 |
|  | Positive - Neutral | 94.6 [90.5–98.7] | p < .05 |
|  | Ambivalent - Negative | 4.1 [0.9–7.3] | p < .05 |
|  | Ambivalent - Neutral | 3.5 [0.0–7.0] | p < .05 |
|  | Negative - Neutral | -0.6 [-1.9–0.6] | p = 0.1689 |
| Love | Positive - Ambivalent | 82.9 [74.6–91.2] | p < .05 |
|  | Positive - Negative | 88.9 [83.3–94.5] | p < .05 |
|  | Positive - Neutral | 90.6 [86.1–95.1] | p < .05 |
|  | Ambivalent - Negative | 6.0 [1.4–10.6] | p < .05 |
|  | Ambivalent - Neutral | 7.7 [3.6–11.8] | p < .05 |
|  | Negative - Neutral | 1.7 [-0.3–3.7] | p < .05 |
| Notes. Entries are percentage-point differences in model-based probabilities with 95% Wald CIs, computed from nnet::multinom via emmeans (type = 'response'). P-values are BH-adjusted within emotions. | | | |

Table S10. Between-emotion contrasts within each valence

| **Valence** | **Emotion** | **Probability [95% CI]** | **CLD* group** |
| --- | --- | --- | --- |
| Positive | Compassion | 42.0 [36.7–47.2] | a |
|  | Commotion | 65.9 [60.1–71.6] | b |
|  | Awe (threat) | 71.0 [65.5–76.5] | b |
|  | Love | 90.6 [87.4–93.8] | c |
|  | Awe (positive) | 91.4 [88.3–94.6] | cd |
|  | Admiration | 91.6 [88.3–94.8] | cd |
|  | Elevation | 94.8 [92.2–97.4] | cd |
|  | Gratitude | 95.3 [92.8–97.7] | d |
| Ambivalent | Elevation | 3.2 [1.2–5.3] | a |
|  | Gratitude | 4.1 [1.8–6.4] | ab |
|  | Admiration | 5.5 [2.8–8.2] | ab |
|  | Awe (positive) | 7.4 [4.4–10.3] | b |
|  | Love | 7.7 [4.8–10.6] | b |
|  | Commotion | 18.5 [13.7–23.2] | c |
|  | Awe (threat) | 24.2 [19.1–29.4] | cd |
|  | Compassion | 30.6 [25.7–35.5] | d |
| Negative | Gratitude | 0.0 [-0.0–0.0] | a |
|  | Elevation | 0.0 [-0.0–0.0] | a |
|  | Awe (positive) | 1.2 [-0.0–2.4] | ab |
|  | Admiration | 1.3 [-0.0–2.6] | ab |
|  | Love | 1.7 [0.3–3.1] | b |
|  | Awe (threat) | 3.8 [1.5–6.0] | b |
|  | Commotion | 15.0 [10.6–19.3] | c |
|  | Compassion | 25.3 [20.7–29.9] | d |
| Neutral | Love | 0.0 [0.0–0.0] | a |
|  | Awe (positive) | 0.0 [-0.0–0.0] | a |
|  | Gratitude | 0.6 [-0.3–1.6] | a |
|  | Commotion | 0.7 [-0.3–1.7] | a |
|  | Awe (threat) | 1.0 [-0.2–2.2] | a |
|  | Admiration | 1.6 [0.1–3.1] | a |
|  | Elevation | 1.9 [0.3–3.6] | a |
|  | Compassion | 2.1 [0.6–3.6] | a |
| Notes. Values are model-based probabilities (%) with 95% Wald CIs (emmeans, type = 'response'). * CLD = Compact Letter Displays. Letters indicate BH-adjusted pairwise differences among emotions within each valence; means sharing a letter are not shown to differ at α = .05. | | | |

*The Influence of Religiosity on Emerging Themes*

Given the predominance of Religious and Spiritual themes that emerged in Study 2, we hypothesized that participants’ level of religiosity could partially explain the high frequency of these themes. Consistent with broader Brazilian demographics, most respondents indicated a religious affiliation, while only 16.7% reported having none (Figure S1A). Additionally, those who identified as religious attributed high importance to religion (Figure S1B). As expected, participants identifying as religious mentioned Religion and Spirituality in their narratives more frequently than those without a religious affiliation (Figure S2). However, the themes of Religion and Spirituality only represented 10.1% of the themes assigned to narratives for religious participants and 2% of the themes assigned to non-religious participants.


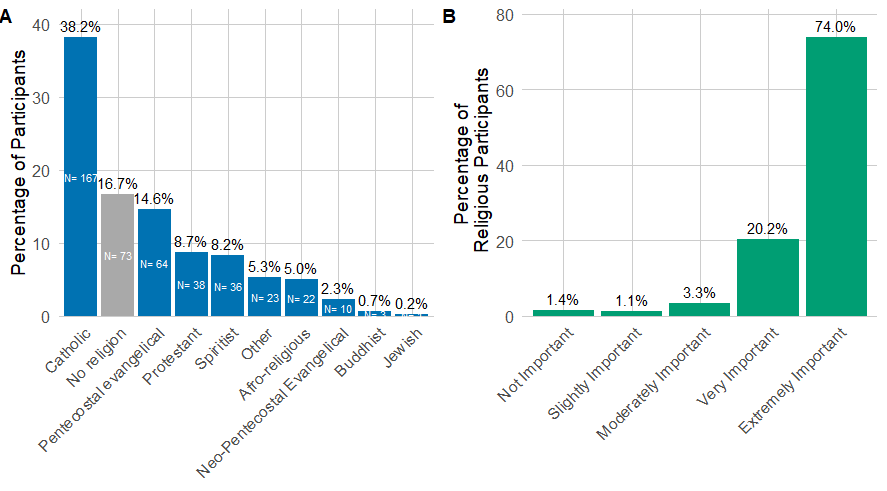


Figure S1. (A) The distribution of participants’ self-reported religious affiliations is expressed as a percentage of the total sample in Study 2. (B) The importance of religion in the lives of religious participants in Study 2.


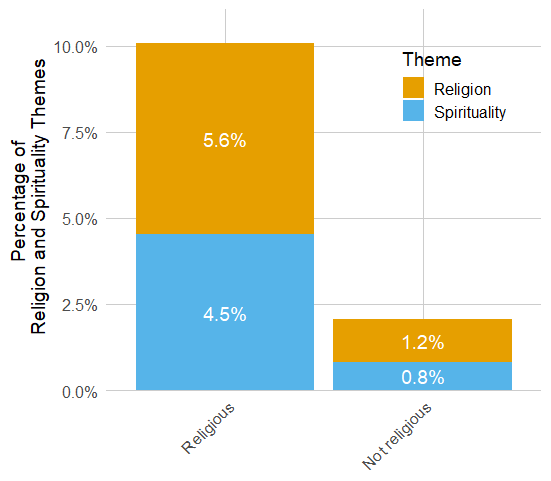


Figure S2. Prevalence of Religious and Spiritual themes in participants’ narratives, categorized by religious versus non-religious status. The height of each stacked segment indicates the relative frequency of each theme in that group. For illustration purposes, only the percentage of themes related to religion and spirituality is shown.
